# A dataset of emissions and removals from scenarios and pathways within long-term national climate strategies – the LTS-SP dataset

**DOI:** 10.1038/s41597-025-04804-4

**Published:** 2025-03-23

**Authors:** Harry B. Smith, Naomi E. Vaughan, Johanna Forster

**Affiliations:** 1 School of Environmental Sciences, University of East Anglia, Norwich, NR4 7TJ UK; 2 Tyndall Centre for Climate Change Research, University of East Anglia, Norwich, NR4 7TJ UK; 3 School of Global Development, University of East Anglia, Norwich, NR4 7TJ UK

**Keywords:** Climate-change policy, Energy policy, Climate-change mitigation

## Abstract

Long-term low emission development strategies (LT-LEDS), supported by Article 4, paragraph 19, of the Paris Agreement, present scenarios and pathways aligned with national long-term climate targets. There is a growing interest in understanding whether the collective effort of national climate plans align with the goals of the Paris Agreement, alongside the feasibility, sectoral focus, and the balance of emissions and removals seen in national scenarios. Here we introduce the long-term strategy scenarios and pathways (LTS-SP) dataset, a dataset presenting scenarios and pathways detailed within LT-LEDS or similar long-term strategies. We detail the level of total and sectoral greenhouse gas emissions in 2050, or the year in which net zero is achieved, alongside the emissions and removals from land-use, land-use change, and forestry (LULUCF) and removals from engineered carbon dioxide removal (CDR) methods. We provide a comprehensive overview of our procedure and compare our dataset with current published estimates. We end by summarising several caveats to our dataset, detailing the limitations of LT-LEDS, and their use in climate policy research.

## Background & Summary

Current national climate policies to address greenhouse gas (GHG) emissions are insufficient to reach the goal of the Paris Agreement, to limit global average temperatures to well below 2 °C above pre-industrial levels, pursuing efforts to limit the increase to 1.5 °C^1^. Despite the ‘*ambition gap*’ that remains, the ratification of the Paris Agreement has led to a groundswell of national net zero targets^2^, near-term climate pledges^3^, and multilateral initiatives, aiming to further spur climate action^4^. At the end of 2024, 147 countries have adopted a form of net zero or neutrality target^5^, 168 nationally determined contribution (NDCs) have been submitted^3^, and international climate initiatives total over 500^6^.

Climate change, however, is a ‘*long problem*’, requiring, in turn, long-term policy-planning, coordinating policies across multiple sectors of an economy towards a long-term goal^7,8^. Given the gradual diffusion of technologies and policies, this is an effort that must be sustained for multiple decades, suggesting a decisive role for national governments^9,10^. For national governments long-term planning can involve creating strategies to guide policy and investment, setting out a strategic direction and anticipating future needs^8^. Article 4, paragraph 19, of the Paris Agreement supports these efforts by inviting parties to ‘*strive to formulate and communicate long-term low emission development strategies*’ (LT-LEDS), strategies that integrate climate mitigation, climate adaptation, and economic development, through a long-term planning process, extending to the mid-century or beyond^11,12^. Concurrent with the rise of national net zero targets, therefore, has been a rise in the submission of LT-LEDS and similar long-term strategies (LTS).

The concept of LTS in climate negotiations precedes the Paris Agreement, and dates to COP15 (Conference of the Parties), held in Copenhagen in 2009. The Copenhagen Accord, agreed at COP15, recognised ‘*that a low-emission development strategy is indispensable to sustainable development*’ (Decision 2/CP.15, paragraph 2) but provided no definition as to what these strategies should entail, nor any explicit calls for their development. The Cancun Agreements, reached the following year at COP16, improved upon COP15, by further deciding ‘*that developed countries should develop low-carbon development strategies or plans*’ (Decision 1/CP.16, paragraph 45) and encouraging *‘developing countries to develop low-carbon development strategies or plans in the context of sustainable development*’ (Decision 1/CP.16, paragraph 65).

The 2015 Paris Agreement, adopted at COP21, formalised these efforts as LT-LEDS, inviting these strategies to be communicated by 2020, with a focus on the mid-century, 2050 (Decision 1/CP.21, paragraph 35). LT-LEDS have routinely featured in the COP decisions that followed. The Katowice Climate Package agreed at COP24, in 2018, reiterated the 2020 deadline (Decision 1/CP.24, paragraph 21). The Glasgow Climate Pact, agreed at COP26 in 2021, urged countries yet to submit their LT-LEDS to do so before the next COP, and called for submitted LT-LEDS to be updated according to the best available science (Decision 1/CMA.3, paragraphs 34 & 35) [CMA referring to decisions from Parties to the Paris Agreement, CP referring to Parties to the United Nations Framework Convention on Climate Change, or UNFCCC]. This same request was made in the Sharm el-Sheikh Implementation Plan, agreed at COP27 (Decision 1/CMA.4, paragraphs 24 & 25), and the conclusion of the outcome of the first Global Stocktake at COP28 in 2023 (Decision 1/CMA.5, paragraphs 20, 40, and 42).

Unlike NDCs, LT-LEDS are not mandatory, and there is no specific guidance from the UNFCCC on how they should be structured and what they should entail. COP22 in 2016, however, announced the 2050 Pathways Platform, an initiative intended to support the development of LT-LEDS, under which informal guidance is produced^13^. Despite a lack of formal guidance, LT-LEDS are formally part of the input that informs the Global Stocktake within the Paris Agreement, the process carried out every five years through which the collective progress of parties to the agreement is assessed (Decision 19/CMA.1, paragraph 36[a]). LT-LEDS, therefore, will likely continue to play a formative role within the UNFCCC, as parties update their NDCs for 2035, during 2025 (Decision 1/CP.21, paragraph 25).

Climate policy research has to date principally focused on NDCs, given their prominence within the Paris Agreement and the need to align climate ambitions with the 2030 benchmarks mainstreamed in IPCC Assessment Reports^14,15^. LT-LEDS, however, have recently gained momentum in climate policy research. LT-LEDS have been used to; refine the projected global temperature outcomes of national pledges and targets^15^, explore the credibility of national net zero targets^16^, and examine the remaining carbon budget^12^, demonstrating their relevance to assessing the ambition of climate policy. LT-LEDS have similarly proved valuable in assessing the plans of national governments towards certain low-carbon technologies or sectors. LT-LEDS have been used to explore the degree to which domestic climate policy directly targets fossil fuel production^17,18^ and to examine the role of geological storage^19^. Multiple articles examine the role of carbon dioxide removal (CDR), largely owing to the more prominent role of CDR in compensating for residual hard-to-abate emissions at the point of net zero, coinciding with the focus of LT-LEDS on the mid-century^20–23^.

Scenario or pathway data from LT-LEDS has been included in several prominent global assessments, including the 2023 United Nations Environment Programme (UNEP) Emissions Gap Report^24^, the 1^st^ and 2^nd^ edition of the State of Carbon Dioxide Removal Report^25,26^, and the UNFCCC LT-LEDS Synthesis Report^27,28^, in addition to estimates across academic and grey literature^22,29^. This presents a need for a standard dataset supported by a common methodology. This dataset should be detailed in its coverage, methodology, and limitations, to aid reproducibility and serve as a resource for climate policy research.

This data descriptor serves as an initial effort, documenting in detail the methodology for producing a consistent dataset of GHG emissions and removals from scenarios and pathways contained within long-term strategies (LTS) – the long-term strategy scenarios and pathways (LTS-SP) dataset. The dataset assesses scenarios and pathways against long-term targets detailed within LTS, and details emissions and removals in 2050, and the date of net zero GHGs, using a consistent definition of sectors and categorisation of CDR methods. It concludes by validating the dataset against estimates in academic and grey literature.

## Methods

The method has been developed to be reflexive to the detail presented across available LTS, based on our own previous analysis^30,31^. We first describe the source and nature of the strategies, then we detail our procedure for analysing elements contained therein. We combine two sources of long-term strategies, LT-LEDS submitted to the UNFCCC Secretariat, and European Union (EU) long-term strategies (EU LTS), submitted to the European Commission. The dataset will be updated periodically at the repository listed in ‘*Data Records*’, as LT-LEDS or EU LTS are revised or newly published. At the time of writing, January 2025, we note several LT-LEDS in development, including strategies for Jamaica, Jordan, Kenya, Mozambique, and Pakistan^32–36^.

### Long-term strategies

The LTS-SP dataset contains 91 long-term strategies in total, of which 11 have been superseded by revised submissions, leaving 80 ‘*active*’ LTS. Of the 80, 74 are LT-LEDS submitted to the UNFCCC Secretariat and six are EU LTS submitted to the European Commission.

We analysed all 85 strategies made available by the Secretariat on the UNFCCC’s long-term strategies portal (ref. ^37^), published prior to or during COP29, held in November 2024. The long-term strategies portal details all current submissions by parties available in a UN language (Arabic, Chinese, English, French, Russian and Spanish), alongside previous submissions for countries that have since revised or updated their submission. Official and unofficial translations are also made available. Notably, LT-LEDS are far fewer than NDCs, with the latter submitted by all 195 parties^3^.

LT-LEDS nevertheless cover a critical mass, with long-term strategies covering 74% of 2023 GHG emissions (including land-use)^38^ and 85% of global gross domestic product (GDP)^39^. The 2023 LT-LEDS Synthesis Report estimates a similar coverage, assessing that LT-LEDS communicated by parties prior to 25^th^ September 2023, cover 76% of total global greenhouse gas emissions in 2019 (excluding land-use), 87% of global GDP, and 68% of the global population^28^.

There is substantial overlap between LT-LEDS and the EU LTS developed by EU Member States. 17 of the LT-LEDS submitted by EU Member States have dual status, serving also as the Member State’s EU LTS. Given the dual role of these LT-LEDS, and their common purpose, it is reasonable to consider those Member State strategies that have not been submitted to the UNFCCC, but have been submitted to the European Commission, as equivalent to LT-LEDS.

EU LTS for Member States are made publicly available on the European Commission’s portal for national long-term strategies (ref. ^40^). Article 15 of the Regulation on the Governance of the Energy Union and Climate Action (EU/2018/1999) sets out a process for the Member States to prepare these strategies in a manner aligned with their commitments under the UNFCCC and the Paris Agreement. Both reporting exercises therefore share a common purpose. We include the EU LTS for six Member States within our dataset, leading to a total of 80 active strategies (excluding the EU’s combined strategy). We further cross-check strategies for all Member States between the European Commission’s portal for national long-term strategies and the UNFCCC’s long-term strategies portal. In cases where differing strategies can be found on both portals, we prioritise the version found on the UNFCCC’s portal. We exclude the LT-LEDS for the EU, on the basis that it is supranational in scope. For all strategies, we prioritise English translations if available for non-English strategies and machine translate remaining strategies using translation software. To indicate that our dataset contains strategies from multiple sources, we use the term long-term strategies, abbreviated to LTS, throughout. We revert to using either LT-LEDS or EU LTS if referring to their reporting, or in reference to a specific national strategy.

Submissions to either portal were made around key dates, such as COPs or deadlines in legislation or COP decisions. The vast majority, 58 of the 80 active strategies, were recently published between 2021–2024. Geographically, the majority of the active strategies have been submitted by countries in Europe (36) and Asia (17), with only nine from African states and four from South America. The 11 countries that have revised and updated their strategies tend to similarly be from Europe (4) and Asia (3). Nearly all active strategies (65) are published in English or otherwise have English translations readily available.

**Fig. 1:**
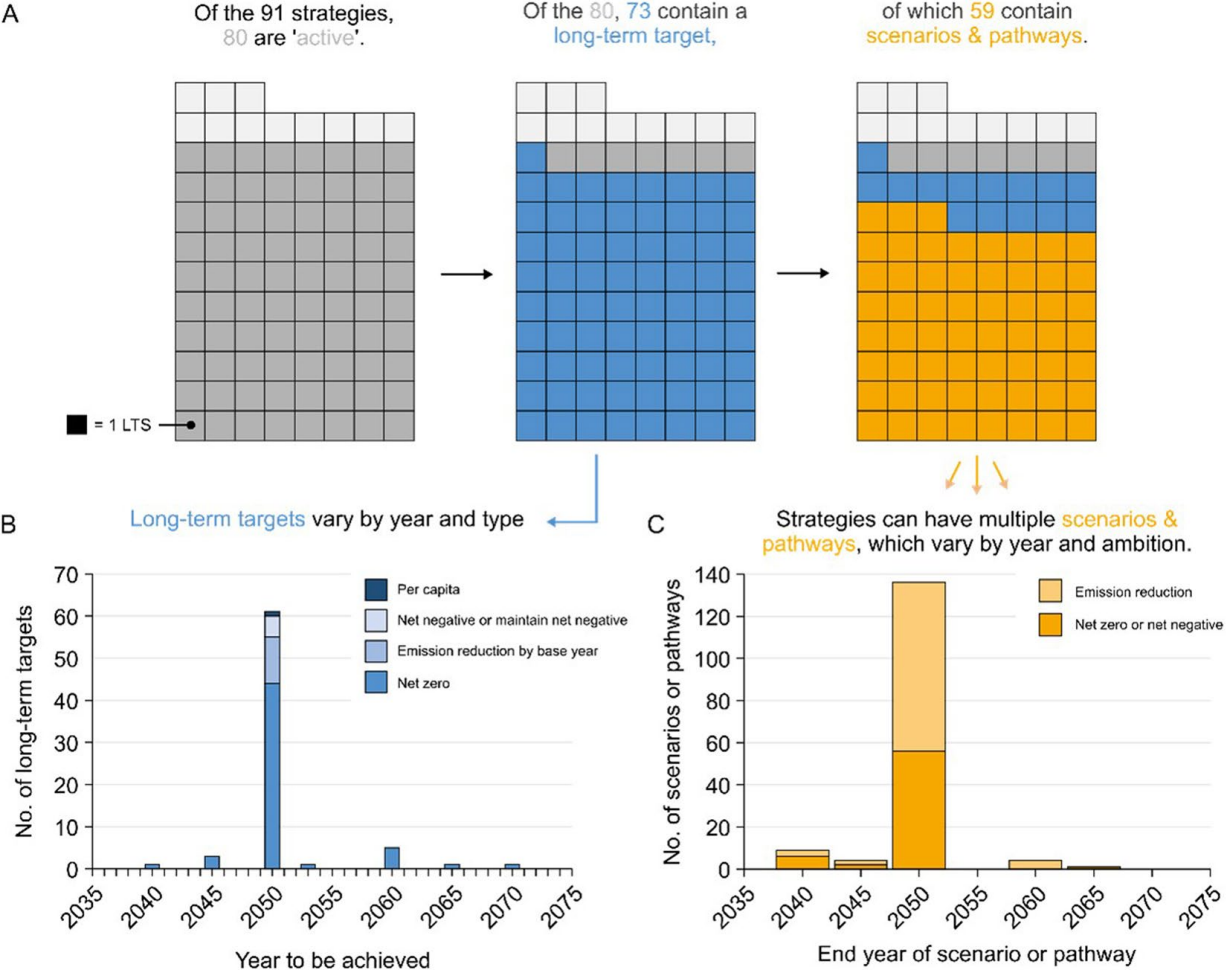
Explanatory figure detailing the contents of the LTS-SP dataset. (**A**) Plot series depicting the number of strategies that contain a long-term target (blue), or scenarios and pathways (yellow). Each block represents a long-term strategy covered by the dataset (grey). (**B**) Bar chart of long-term targets, by year to be achieved, for example, net zero GHGs by 2050. (**C**) Bar chart detailing the number of scenarios or pathways, shown by end year. End year refers to the last available year within the strategy for which data is available.

Once collated, we analyse each strategy according to two main elements, long-term targets, and scenarios or pathways. Of the 80 active strategies currently analysed, 73 contain a long-term target (Fig. 1A). Of those 73 strategies, 59 detail at least one scenario or pathway extending beyond their NDC. Strategies often contain multiple scenario or pathways, meaning, for the 80 active strategies, the LTS-SP dataset details 153 scenarios or pathways from 59 countries (Fig. 1C).

### Long-term targets

Long-term targets are those targets that extend beyond the time horizon communicated in NDCs, such as net zero targets in and around the mid-century. These targets are communicated within LT-LEDS but are often ambiguous in their design as to their precise coverage of sectors and GHGs. This is a widely recognised problem that adds undue uncertainty to the assessment of national pledges and targets^41–43^. Beyond metadata, such as the publication date of the strategy (see ‘*Metadata*’ in Table 2, Data Records), long-term targets are the initial element extracted from LTS. We identify several elements that impact upon the definition of a long-term target, and therefore scenarios and pathways that meet these targets.

**Fig. 2:**
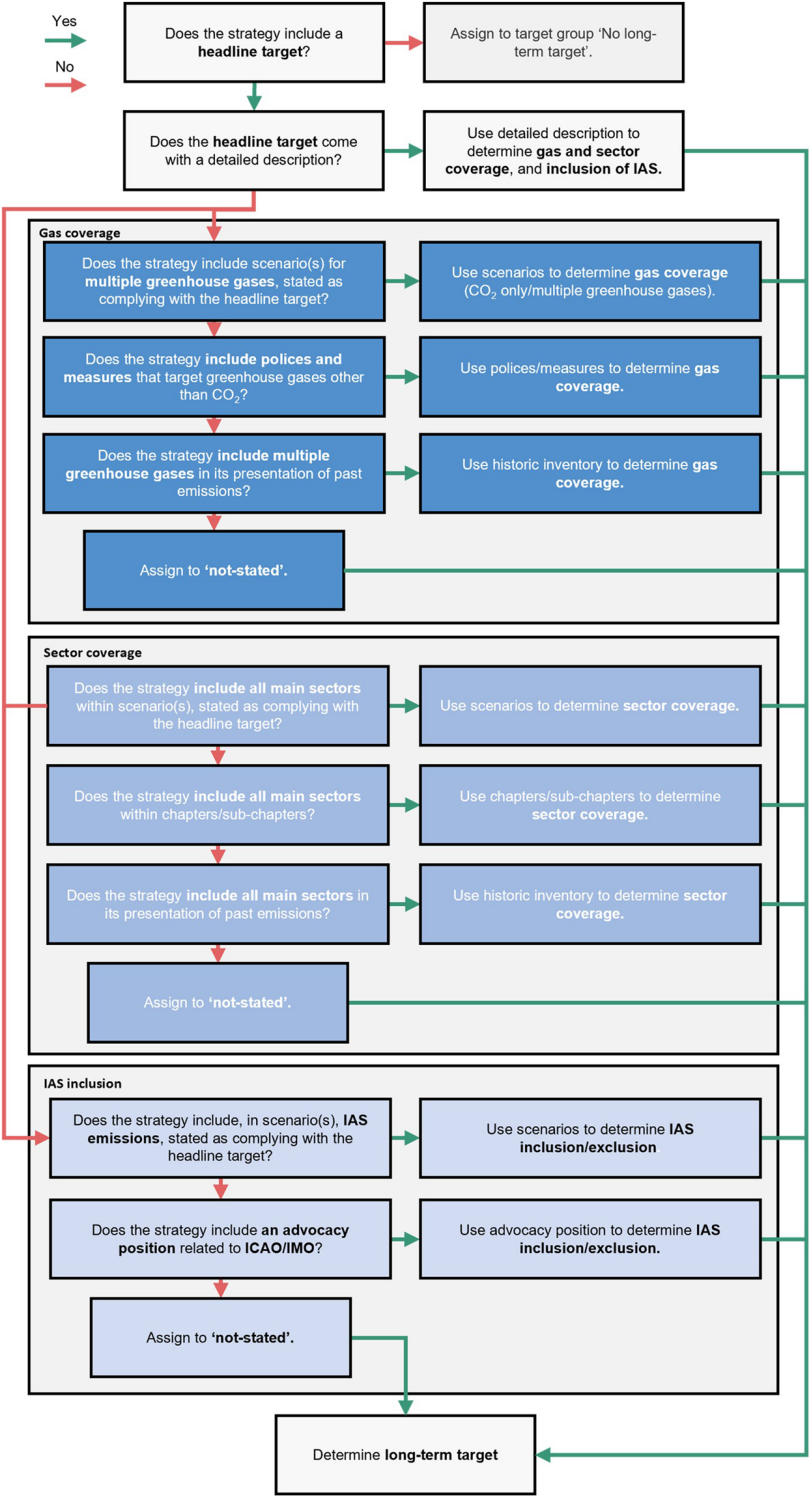
Analytical procedure for long-term targets. Shown as a decision chart. IAS refers to international aviation and shipping. ICAO and IMO refer to the International Civil Aviation Organization and the International Maritime Organization respectively. If a target definition includes only one or two elements, for example, the gas coverage and the inclusion or exclusion of IAS, the outstanding element is determined by the relevant section of the decision chart, in this example, the sector coverage.

We differentiate between the ‘*headline target*’, describing the target as it is written and referred to within the country’s strategy, and a ‘*long-term target*’, which we define as the headline target amended for its gas coverage. For example, a headline target of ‘*carbon neutrality*’ that covers the main greenhouse gases (carbon dioxide [CO_2_], methane [CH_4_], and nitrous oxide [N_2_O]), is best described as a long-term target of ‘*net zero GHGs*’, as ‘*carbon neutrality*’ implies the consideration of only CO_2_^44^. We identify the headline target within the strategy, alongside any description clearly stated. We then confirm the timing of the target, the gas and sector coverage, alongside any inclusion of emissions from international aviation and shipping (IAS)^45^, either via the description stated or by a series of ordered indicative elements detailed in Fig. 2. We define these terms in Table 1. Collating and understanding long-term targets are necessary for two related reasons, firstly to clarify the headline target, which may be miscommunicated if interpreted as written, and to prioritise scenarios and pathways that can be demonstrated to comply with the target.

**Table 1:** Definitions of terms relevant to long-term targets.

Term	Definition
Headline target	The country’s long-term target as it is written and referred to within the country’s strategy.
Long-term target	The headline target amended for its *gas coverage*.
Gas coverage	The GHGs covered by the long-term target. For example, the target may include only carbon dioxide (CO_2_) or include, in addition, the other two main greenhouse gases, methane (CH_4_) and nitrous oxide (N_2_O).
	Similarly, it may cover the ‘*Kyoto basket*’ of gases covered by Annex A of the 1997 Kyoto Protocol, including the three main greenhouse gases, plus hydrofluorocarbons (HFCs), perfluorocarbons (PFCs), and sulphur hexafluoride (SF_6_). It may also include, in addition, nitrogen trifluoride (NF_3_), which was added in the 2012 Doha Amendment to the Kyoto Protocol. These seven gases continue to be reported under the Paris Agreement, see Decision 18/CMA.1, Annex, paragraph 48.
	We differentiate between the consideration of CO_2_ only (for example, net zero CO_2_), or the consideration of multiple greenhouse gases, implying coverage of at least CO_2_, CH_4_ and N_2_O (for example, net zero GHGs).
Sector coverage	The sectors covered by the long-term target. Sector coverage refers to the inclusion or exclusion of all main sectors, including Energy, Transport [independently or as a subsector of Energy], Industry, Agriculture, Waste, and LULUCF, based upon the 2006 IPCC Guidelines for National Greenhouse Gas Inventories (hereafter the 2006 IPCC Guidelines).
	This includes subsectors, sources, or combinations thereof, for example Agriculture, Forestry, and Other Land Uses (AFOLU).
IAS inclusion	The inclusion or exclusion of emissions attributable to the country from international aviation and shipping (IAS). Domestic aviation and shipping, emissions from passenger or freight traffic that departs and arrives in the same country, is typically included in national greenhouse gas inventories and therefore long-term targets set, unless otherwise stated^47^.
	IAS is typically estimated based on fuel sold, or based on ‘*bunker fuels*’, but is excluded from national totals and reported separately within national greenhouse gas inventories, and therefore commonly excluded from long-term targets^47^.

**Fig. 3:**
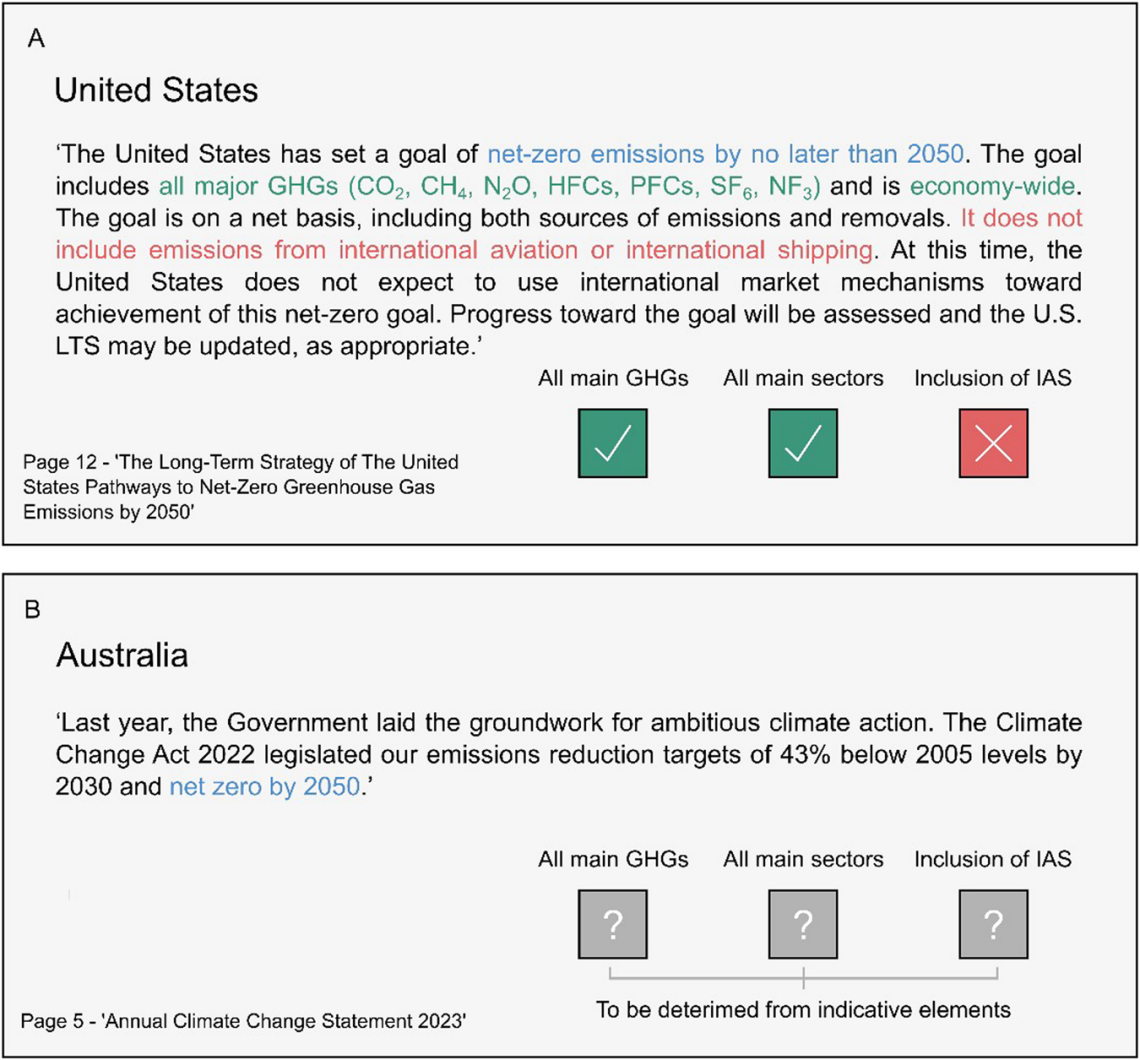
Examples of headline targets from strategies. (**A**) provides an example from the United States’ LT-LEDS, detailing the headline target (blue) alongside a detailed description further specifying the gas coverage (green), sector coverage (green) and exclusion of IAS (red). (**B**) provides an example from Australia’s LT-LEDS, which provides only a headline target without a detailed description. As a result, the gas coverage, sectors coverage and inclusion of IAS are to be determined from indicative elements shown in Fig. 2.

In Fig. 2, the indicative elements are ordered in terms of priority. For example, an LTS may include a detailed definition of its headline target, following best practice^12^. If this definition provides the necessary detail to determine the coverage of the target, this is used. For example, the LT-LEDS for the United States, ‘*The Long-Term Strategy of the United States*’, includes a detailed target description, specifying both the gas and sector coverage, as well as the exclusion of IAS and the current exclusion of the use of international offsets (see Fig. 3A). Strategies, however, may not provide this necessary detail, for example Australia’s LT-LEDS, simply describes its headline target as ‘*net zero by 2050*’, without reference to coverage or inclusion (see Fig. 3B). In these cases, it is necessary to discern coverage by other indicative elements (see Fig. 2). Multiple indicative elements may be used to determine coverage for strategies that are unclear.

The 2006 IPCC Guidelines are the main reference for the development of National Greenhouse Gas Inventories (NGHGIs) - detailed national datasets of emissions by sources and removals by sinks^45^. The guidelines provide the naming convention for emission sources and removals categorised into a nested structure of national sectors^45^. NGHGIs are commonly the basis of scenarios and pathways included within strategies, as well as the basis for tracking trends in historical emissions, and therefore serve as the accounting logic for sectors, unless altered to better suit national circumstances. We take the approach advocated by the LT-LEDS Synthesis Report, analysing, if not explicitly specified in a target description or scenario or pathway modelling, the coverage of sectoral chapters as indicative of the sector coverage^28^.

Caution should be exercised for non-Annex I countries, which submit NGHGIs only periodically as opposed to an annual basis^46^. As a result, NGHGIs tend to be more limited for non-Annex I countries^46^, meaning countries may be yet to source the necessary activity data to estimate emissions for a certain sector, impacting upon the inclusion of the sector within scenarios or pathways. This should not necessarily be considered as an omission of the sector from the long-term target, as improvements in inventory capacity may lead to the estimation of emissions in future NGHGI reporting or in revisions to the LT-LEDS. Similarly, some sectors may not occur within an economy, for example, states with limited industry may not report industrial emissions^47^. Sectors ‘*not occurring*’ should not necessarily be seen as excluded from the long-term target.

For international aviation and shipping, the inclusion of emissions attributed to a country may result in the need for increased removals or further emission reductions to achieve a set long-term target (for example, as observed in the UK LT-LEDS^48^). Typically, emissions from IAS are considered under international organisations, such as the International Civil Aviation Organization (ICAO) and the International Maritime Organization (IMO). States participate in these organisations to multilaterally coordinate climate policy owing to the cross-border nature of emissions^49,50^. Nevertheless, NGHGIs typically estimate emissions from IAS on a ‘*bunker fuel*’ basis, that is emissions from fuel sold during refuelling for international journeys^51^. These emissions are, however, ‘*memo items*’, excluded from national totals and therefore, by extension, long-term targets^47,51^. Select countries, such as the United Kingdom, include IAS emissions within their long-term target, net zero GHGs by 2050^48^. Inclusion does not necessarily imply unilateral action on these emissions but can be complementary to international efforts, and a recognition that meeting the Paris Agreement for IAS will require new infrastructures and industries within national borders, such as engineered CDR and new synthetic fuels^51^. The inclusion of IAS is therefore likely explicit, if a target description is included, or implied by supporting scenario or pathway modelling. As a last resort, the inclusion or exclusion of IAS can be inferred by the policy position, if stated, of the country towards the ICAO or IMO. In cases where no mention of IAS is found within the strategy, it is likely that the country follows existing practice, which is to exclude these emissions from scope.

### Emissions & removals within scenarios and pathways

**Fig. 4:**
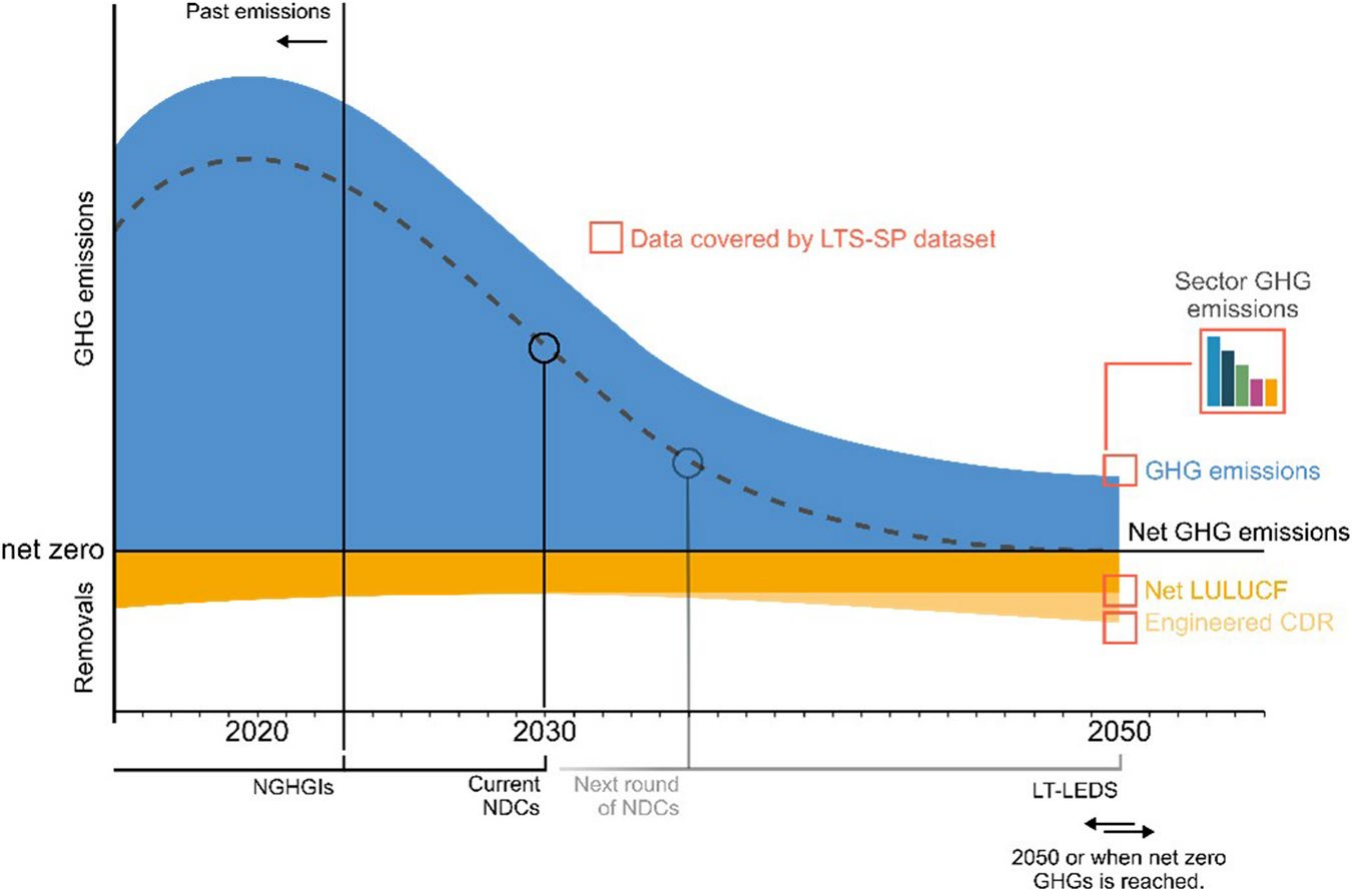
Stylised net zero pathway showing the data covered by the LTS-SP dataset. Boxes in red are data points covered by the LTS-SP dataset. Headers along the dateline show the years covered by different reporting obligations, including current NGHGIs (2022, soon 2023), current NDCs (2030), and new NDCs due in 2025 (2035). Figure based on Fig. 2, Cross-Chapter Box 8, IPCC AR6 WGIII (ref. ^21^). Figure depicts a stylised pathway that reaches net zero GHGs in 2050. Alternative pathways are possible and depend on the scenario or pathway design, long-term target, and timing.

Once long-term targets are collated, we identify all instances of scenario and pathway modelling found within the strategies. This requires a reflexive approach, adaptive to the detail presented, as LTS can include no modelling, present only select results, such as total emissions, or present emission estimates across multiple sectors. We employ the following approach, we first identify whether the strategy includes scenarios or pathways that extend beyond the country’s NDC (for example, 2030). If so, we collate the total GHG emissions excluding LULUCF, any estimates of sectoral emissions, net LULUCF emissions, and any removals from engineered CDR methods, detailed in data tables, text, or legible graphs and figures (Fig. 4). Scenarios are based on a key set of modelled assumptions made in an LTS, relating to, for example, the use of specific low-carbon technologies. Pathways, meanwhile, can take the form of a series of policy targets, prescribing a pathway for GHG emissions over time, or a range informed by multiple scenarios. Pathways tend to have a less direct connection to the assumptions underpinning them. If the strategy includes emissions by sector or by subsector or source, we collate these estimates, cross-checking the sum against total emissions excluding LULUCF (Fig. 4).

**Fig. 5:**
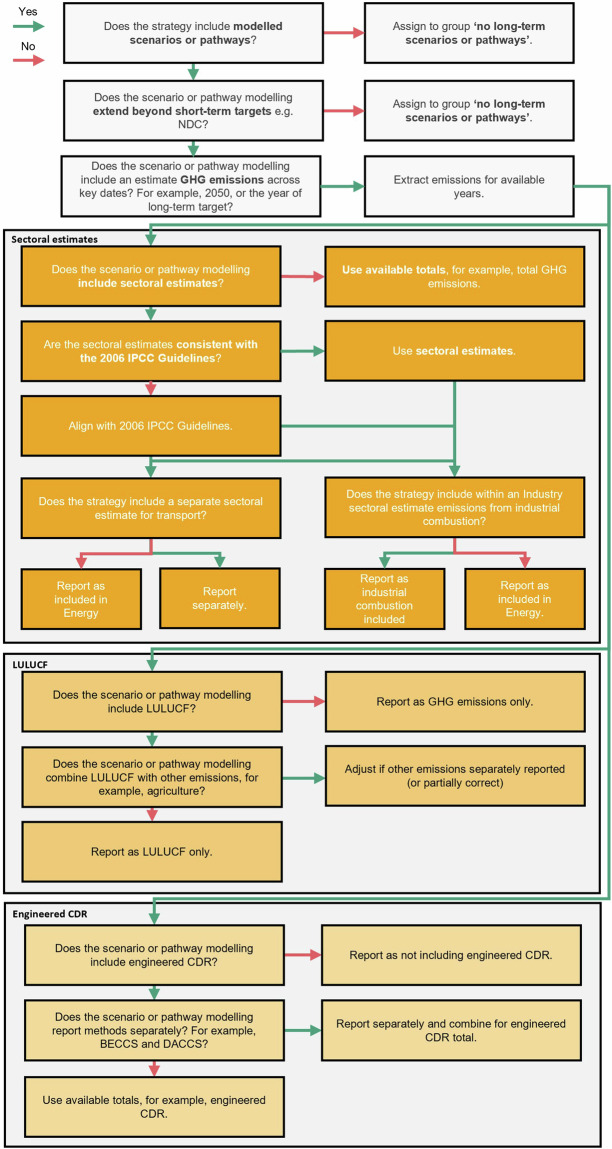
Analytical procedure for modelled scenarios or pathways. Shown as a decision chart. We also include a check to verify whether the scenario or pathway modelled complies with the long-term target from Fig. 2.

We prioritise the year 2050, owing to its alignment with existing national net zero targets and its importance as a milestone within scenarios assessed by the IPCC^52^. This is done mindful of the long-term target. For example, if the long-term target is net zero CO_2_, scenarios and pathways may detail only CO_2_ emissions, meaning it is necessary to make assumptions around the level of non-CO_2_ emissions, unless otherwise specified. Similarly, if the long-term target extends beyond 2050, or is to be reached prior, we assess emissions and removals at the point net zero GHGs is achieved. Each step of this approach is shown in Fig. 5.

Unlike reporting obligations within the UNFCCC and elsewhere in the Paris Agreement, LT-LEDS are not associated with guidance that recommends a specific scenario logic. Adopted guidelines within the UNFCCC, recommend a logic of three projections, (i) ‘*with measures*’, (ii) ‘*with additional measures*’ and (iii) ‘*without measures*’ or the ‘*baseline*’ (for example, the annex of Decision 18/CMA.1). A ‘*with measures*’ projection encompasses currently implemented and adopted policies and measures, whilst a ‘*with additional measures*’ projection includes planned policies and measures yet to be adopted. This structure of projections is designed to identify the emission gap remaining between current or planned policy efforts and near-term climate targets, such as NDCs. Many LT-LEDS therefore adopt a similar structure for scenarios or pathways, but also include scenarios or pathways that seek to identify the conditions under which a long-term target may be met^53^. Multiple scenarios or pathways may be included, designed to explore a range of technological options or policy approaches. Given the absence of formal guidelines for LT-LEDS, strategies may include multiple types of scenario, with only a select subset achieving the long-term target. We do not classify scenarios by the extent of adopted or additional policies and measures but by whether they achieve or exceed the long-term target set within the strategy.

The term ‘*residual emissions*’ has recently emerged in climate governance to describe positive emissions remaining at the time of net zero^22,54^. This, however, is but one definition and use of the term, with the term used elsewhere to describe the emissions that remain after an emission reductions target is reached^55^, or the cumulative emissions that continue to be emitted across the century in modelled scenarios assessed by the IPCC^56^. In these examples, ‘*residual emissions*’ is used as a term irrespective of achieving net zero. Given the LTS-SP dataset covers a range of scenarios and pathways, targeting a range of mitigation outcomes, including those that focus only on reducing net emissions as opposed to achieving net zero, we do not universally use the term to describe the total or sectoral GHG emissions covered by the dataset, as this would combine multiple definitions. Residual emissions, within the LTS-SP dataset, are, therefore, only those total or sectoral GHG emission in scenarios or pathways that achieve a long-term target of net zero GHGs, adjusting for the timing of net zero GHGs, if achieved earlier than the long-term target itself.

With limited exceptions, LTS largely fail to specify the global warming potentials (GWPs) used when presenting GHG emissions on a CO_2_-equivalent basis. It is likely that many use GWPs for a 100-year time horizon, based on Working Group 1 of the IPCC’s Fifth Assessment Report (or GWP100 AR5), consistent with existing practice for NGHGIs. The use of the GWPs for a 100-year time horizon from Working Group 1 of the IPCC’s Fourth Assessment Report (AR4) may also be probable for those LT-LEDS published prior to 2019, before COP24 led to the adoption of GWP100 AR5 as the standard for Parties (Decision 18/CMA.1, Annex, paragraph 37).

### Sectoral emissions

If the strategy includes emissions by sector or by subsector or source, we collate these estimates, cross-checking the sum against total emissions excluding LULUCF, if presented, or deriving a total if missing. Select strategies, such as Finland, detail emissions from their scenarios using the Common Reporting Format (CRF), the format of Annex I NGHGI submissions to the UNFCCC, meaning results can be directly mapped to sectors used in NGHGI reporting^57^. Many LTS refer to unconventional sectors, or a combination of sub-sectors and sources, meaning it is necessary to use their descriptions to assign to sectors. These sectors, or combinations thereof, are often inconsistent in the emissions they include, with country’s devising a categorisation that better suits national circumstances, for example, the UK Net Zero Strategy’s ‘*emission taxonomy*’, which differs from the sectors used within the UK’s NGHGI^58^. As with long-term targets, we include all main sectors, including Energy, Transport, Industry, Agriculture, Waste, and LULUCF, based upon the 2006 IPCC Guidelines. We use the reporting tables (Table 8.2, Volume 1, Chapter 8), found within the 2006 IPCC Guidelines, or otherwise the CRF, to align descriptions of sectors or sub-sectors and sources to these main sectors^47,57^.

Care is required in two cases, the inclusion of transport emissions within Energy and the inclusion of energy use emissions in addition to process emissions, within a wider Industry category (see Fig. 5). This is owing to the changing focus of emissions as countries decarbonise and a need therefore to present sectors that are of interest towards the end of decarbonisation, as opposed to current NGHGI practice. Of recent interest are the so-called ‘*hard-to-abate*’ sectors, typically used to describe emissions from long-range transport, such as aviation and shipping; heavy industries, such as steel, cement, and chemicals; and agriculture, for sources of non-CO_2_ emissions, such as those emitted by livestock or from fertilisers^21^. We similarly note a recent interest in the study of residual emissions^22,59^. Though distinct from residual emissions, owing to their difficulty of abatement, emissions from hard-to-abate sources are likely to be residual and therefore indirectly mitigated through CDR, as opposed to directly abated at source^21^. Creating a dataset that is of use to climate policy research should therefore seek to balance current practice with the likely direction of research. We therefore split, where specified, transport emissions from energy, owing to the tendency to describe aviation and shipping emissions as hard-to-abate and residual^21^.

Current practice in the 2006 IPCC Guidelines is to report emissions from transport in an overall Energy sector, owing to the use of national energy balances^47^. Where not possible, for example, when a strategy fails to further disaggregate the Energy sector, we use the notation key ‘*included elsewhere*’ or ‘*IE*’, indicating where emissions are included^47^. Notation keys are common across NGHGI reporting to indicate where emission categories may be incomplete, missing, or inconsistent with NGHGI practice^47^.

The 2006 IPCC Guidelines also advocates the reporting of emissions from industrial fuel combustion within Energy, whereas select countries report this within an Industry sector. This may be desirable as decarbonising industry requires an approach that addresses the thermal heat required for industrial processes, conventionally supplied by the combustion of fossil fuels, in addition to addressing the emissions that arise from chemical reactions directly within the industrial process^60,61^. We adjust for this where possible, to ensure Industry consists of only emissions from industrial processes as opposed to combustion.

### Engineered carbon dioxide removal

We identify estimates of removals from engineered CDR, specifying separate estimates for direct air carbon capture and storage (DACCS) and bioenergy with carbon capture and storage (BECCS), where available. The terminology surrounding CDR is still changing and CDR is commonly conflated with carbon capture utilisation and storage (CCUS)^62^. The overlap between engineered CDR and CCUS is limited in practice, given there are few means of CCUS that satisfy the criteria for CDR, that is, the CO_2_ originates from the atmosphere, the CO_2_ captured is permanently stored and not soon reemitted, and the net quantity of CO_2_ removed is greater than the quantity of GHGs emitted^63,64^. Therefore, though commonly conflated, there is little justification for doing so. Owing to this ambiguity, we make several assumptions when engineered CDR is included, for example, if CCUS is presented as a removal within scenario or pathway modelling, it may be considered as engineered CDR unless otherwise specified as short-term utilisation. We are also alert to unconventional uses of terminology, for example, Finland’s use of ‘*Bio-CCS*’ as an equivalent term to BECCS. Given the current lack of guidance concerning the reporting of engineered CDR^65^, there are instances where removals from CDR methods are included as net emissions or removals within a sectoral total, for example, removals from BECCS are included within the total for the Energy sector. This follows the logic supported by the 2006 IPCC Guidelines, reporting emissions and removals within the sectors in which they occur^47^. This may require assigning a main purpose. For example, the 2006 IPCC Guidelines advocate that, if waste is incinerated for the purposes of energy recovery it should be accounted for in the Energy sector as opposed to Waste^47^. It is therefore logical that if biomass is combusted for energy purposes, it’s removals should be similarly accounted for in Energy. We therefore adjust, where possible, cases where sectoral emissions and removals from engineered CDR are combined. Where not possible, we report what emissions are combined and whether this leads to an underestimate in sectoral or total emissions.

In select cases, only partial corrections are possible. For example, in a case where a sub-sector or sectoral total is net-negative owing to the inclusion of removals from engineered CDR, and the extent of removals from engineered CDR or the emissions excluding removals are not stated, we can infer a minimum bound for engineered CDR by the extent of net-negative emissions. It is likely that this would represent an underestimate. We document these cases as ‘*partial corrections*’. Similarly, if removals cannot be separated in this manner, for example, removals are included but the sector or subsector total is not net-negative, we document these instances.

### Land-use, land-use change and forestry (LULUCF)

For LULUCF, current practice is to report the sectoral total on a net basis, without distinguishing between emissions and removals^47^. Many strategies therefore report LULUCF according to current practice, obscuring the nature and direction of interventions within the land-use sector (for example, declining rates of deforestation or increasing rates of afforestation can both reduce net LULUCF emissions). We identify estimates of LULUCF specifying net emissions. As with engineered CDR methods, we adjust cases where emissions from other sectors and removals are combined. This is most common with ‘*AFOLU*’, or ‘*Agriculture, Forestry and Other Land Use*’, which combines emissions from agriculture with emissions and removals from LULUCF^47^. Where separation is not possible, we report what emissions are combined and whether this leads to an underestimate. Note that LULUCF estimates within NGHGIs may be defined differently to that of the IPCC Assessment Reports (See Usage Notes).

Once all necessary elements have been collated; total or the sum of sectoral emissions, LULUCF and any inclusion of removals from engineered CDR, we compare the net total to the long-term target, establishing whether the modelled scenario or pathway reaches the long-term target set. This is necessary to discern between scenarios, as typically long-term strategies contain multiple scenarios, differentiated by policy ambition or technology choice. These may or may not reach the long-term target set and cannot necessarily be inferred by the title of the scenario alone.

## Data Records

The dataset can be accessed on Zenodo at 10.5281/zenodo.14943904 (ref. ^66^). The dataset is available as a .xlsx file. The .xslx file contains three main tabs; ‘*Metadata*’, ‘*Long-term targets*’ and ‘*Emissions & removals*’, detailed further in Table 2. At the Zenodo DOI, we maintain a change log, detailing additions, edits or corrections since January 2025^66^.

**Table 2:** A summary of the main tabs and contents of the dataset file.

Tab	Summary
Metadata	A table detailing the long-term strategies covered by the dataset, including their date of publication, name, submission, type, language, number of pages, and annex.
	Submission refers to whether the LTS serves as a countries 1^st^ or 2^nd^ submission to either the UNFCCC Secretariat or the European Commission. Type refers to whether the strategy is a LT-LEDS, EU LTS, or strategy with dual status, submitted as the EU Member State’s EU LTS and LT-LEDS.
	Annex refers to whether the strategy is published by an Annex I or non-Annex I country, a division often used by the UNFCCC and is often indicative of the inclusion of scenario or pathway modelling. Annex I countries are required to submit national greenhouse gas inventories annually, and therefore should have the technical capacity for modelled scenarios and pathways.
Long-term targets	A table detailing the headline targets, coverage and resulting long-term targets detailed within LTS covered by the dataset.
	For each element, a page reference is included documenting the relevant source text, graphs, or tables.
Emissions & removals	A table detailing estimates of GHG emissions excluding LULUCF, sector GHG estimates, and emissions/removals from LULUCF and removals from engineered CDR, detailed within LTS covered by the dataset.
	For each element, a page reference is included documenting the relevant source text, graphs or tables.
	How non-conventional sectors, sub-sectors or sources have been assigned to the main sectors is also reported.

21 of the 80 active strategies, do not detail scenario or pathway modelling. 59 of the 80 active strategies contain quantified scenarios or pathways beyond the country’s NDC. Our dataset, as of publication, therefore, covers this amount. 45 active strategies detail sectoral emission estimates and 49 strategies include estimates for LULUCF. Only 14 strategies include an estimate of engineered CDR. The inclusion of different elements by country is summarised in Table S1 found in Supplementary Information. Users of the dataset, however, should refer to the change log found at the Zenodo address (ref. ^66^) for the most recent assessment of elements included.

## Technical Validation

Three datasets are used to validate our results: the Net Zero Tracker^5^, the Supplementary Data of Buck *et al*., (2023) (ref. ^22^), and New Climate Institute’s 2023 Assessment of the G20 Members’ Long-term Strategies (ref. ^29^). For long-term targets, we validate our dataset against the Net Zero Tracker, an online database tracking targets pledged by nations, regions, cities, and companies^5^. The Net Zero Tracker tracks all Parties to the UNFCCC, using a range of submissions by parties, including LT-LEDS. Validating the LTS-SP dataset against the Net Zero Tracker has two purposes; firstly, to ensure the long-term targets are correctly coded, identifying disparities between the datasets, and secondly to provide an estimate as to the extent of which the LTS-SP may no longer reflect the latest long-term targets.

The Net Zero Tracker uses different codes to the LTS-SP dataset, representing the same or similar elements. For example, the Net Zero Tracker collates ‘*end targets*’, which represent the country’s own description of their target from a pre-determined list of codes^67^. This is analogous to the headline targets within the LTS-SP dataset. In contrast to the LTS-SP dataset, the Net Zero Tracker does not correct the end target for gas coverage, as we do for the headline target to produce the long-term target. We therefore produce a comparative long-term target by combining fields of the Net Zero Tracker dataset. We consulted the fields for ‘*end target*’, ‘*end target year*’, ‘*gasses coverage*’, ‘*international aviation*’, and ‘*international shipping*’. The Net Zero Tracker does not contain a field for sector coverage but does provide a summary of considerations not captured by other fields in the ‘*coverage notes*’ field. Similarly, the Net Zero Tracker contains the ‘*target notes*’ field, which provides further detail relating to the target not captured in other fields.

We compare the long-term targets within the LTS-SP dataset to the Net Zero Tracker, determining whether there are substantive differences between the coverage or nature of the target. For example, with the inclusion of IAS, if the Net Zero Tracker states that these emissions are included, whilst the LTS-SP details that these are excluded or not stated, this would constitute a substantive difference. The Net Zero Tracker stating these emissions are ‘*not specified*’ and the LTS-SP dataset detailing that these emissions are excluded would not constitute a substantive difference. Similarly, disparities in the timing or ‘*end target year*’, or the nature of the target would constitute as substantive, provided these are not modified by the ‘*gasses coverage*’ field in the Net Zero Tracker. That is to say that an end target of ‘*carbon neutral(ity)*’ in the Net Zero Tracker would be considered as equivalent to a long-term target of ‘*net zero GHGs*’ in the LTS-SP dataset, if the ‘*gasses coverage*’ field in the Net Zero Tracker reads ‘*Carbon dioxide and other GHGs*’. If the ‘*gasses coverage*’ reads ‘*Carbon Dioxide only*’ this would be considered a substantive difference. In this manner we capture only key disparities, not simply differences in coding and method. We evaluate disparities across four elements: the nature of the target (for example, net zero GHGs versus an emission reduction target), target year, gas coverage, and the inclusion of IAS. Given the Net Zero Tracker provides no systematic assessment of sectors, we do not compare the datasets according to sector coverage.

**Fig. 6:**
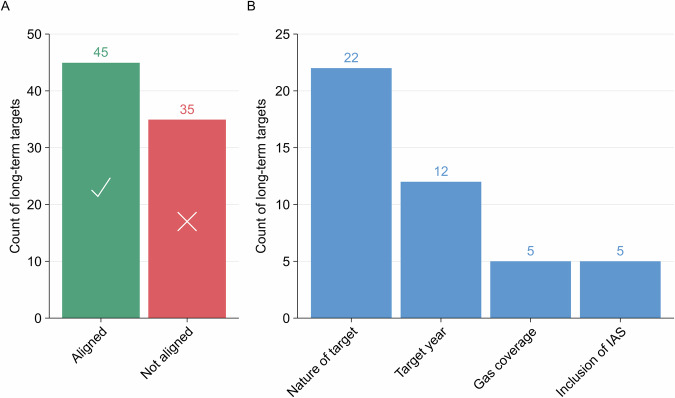
Comparison of long-term targets between the LTS-SP dataset and the Net Zero Tracker. (**A**) compares the alignment or misalignment between the datasets. If ‘*aligned*’, the long-term target in the LTS-SP dataset is not substantively different from the combination of fields assessed in the Net Zero Tracker. If ‘*not aligned*’, the long-term target in the LTS-SP dataset is substantively different on one or more of the four elements assessed in panel B.

We observe a large disparity between the datasets, with long-term targets for 35 of the 80 countries assessed not aligned on at least one or more of the four elements assessed (Fig. 6A). For 22 countries, long-term targets differ according to the nature of the target (Fig. 6B). We investigated all instances. In seven instances, we assess there is no headline target and therefore no long-term target to be assessed, whereas, in five of these cases, the Net Zero Tracker uses the wording of the NDC as the ‘*end target*’. In the remaining two cases, it is not clear how the Net Zero Tracker has arrived at a different target to the long-term target in the country’s LT-LEDS. In six further instances, the long-term target appears to have been upgraded since the publication of the LTS, by for example, introducing a net zero target as opposed to an emission reduction target. In four instances, we determine the long-term target as a variation of a net-negative target, such as maintaining or increasing net-negativity for GHGs. In these cases, the Net Zero Tracker describes these as net zero. 12 countries differ on timing, similarly linked to cases where we assess there is no headline target and the Net Zero Tracker uses the wording of the NDC (five of the 12 cases).

Long-term targets for five countries differ according to their gas coverage. For example, for Nepal, the Net Zero Tracker lists a target of net zero applying to ‘*Carbon dioxide and other GHGs*’, referencing Nepal’s LT-LEDS as a source. We assess this long-term target to apply to only CO_2_ based on the scenarios detailed in the LT-LEDS^68^. In three of these cases, we document exemptions of certain gases, such as methane, from long-term targets, whereas the Net Zero Tracker does not differentiate these elements.

Long-term targets for five countries differ on the inclusion of IAS. In three cases the inclusion of IAS has been conflated with the mention of international targets. This is not comparative to the inclusion of IAS as defined in Table 1.

For emissions and removals, we validate our dataset against the results available in the Supplementary Data of Buck *et al*., 2023, ‘*Why residual emissions matter right now*’ (ref. ^22^). This includes scenario data for 28 LT-LEDS, 18 from Annex I countries and 10 from non-Annex I countries, submitted to the UNFCCC Secretariat prior to mid-2022. Buck *et al*., 2023 focuses on low residual scenarios, presenting the scenario with the lowest residual emissions where multiple scenarios are detailed within strategies, whereas our dataset includes all long-term scenarios found within LTS. We therefore compare the minimum residual emission scenario within our data to the data presented in Buck *et al*., 2023. In select cases, Buck *et al*., 2023 has prioritised those pathways that comply with a set long-term target. For example, if a net zero target is the headline target for the country, scenarios that fail to reach this target are dismissed. We therefore use the page references and target timing within Buck *et al*., 2023’s Supplementary Data to determine how the total is reached and align the data with our own, comparing like with like scenarios where possible. Owing to the publication date of Buck *et al*., 2023, scenarios are often sourced from LT-LEDS that have since been revised or superseded. Given that our intention is to validate the method through which estimates are reached, not compare how scenarios have changed between LT-LEDS, we compare scenarios from past LT-LEDS, even if since revised and superseded by a new submission.

We also compare our data to the data compiled for New Climate Institute’s 2023, ‘*Assessment of the G20 Members’ Long-term Strategies*’ (ref. ^29^), which collates residual emissions, LULUCF and engineered CDR in 2050 for the G20 states. New Climate Institute’s assessment includes both a maximum and a minimum for each element, for example, both maximum and minimum residual emissions. Here residual emissions are not necessarily those at the point of net zero, but rather remaining emissions in 2050 excluding LULUCF or engineered CDR methods. We therefore compare this data to the minimum and maximum found within our data, where a country presents multiple scenarios.

**Fig. 7:**
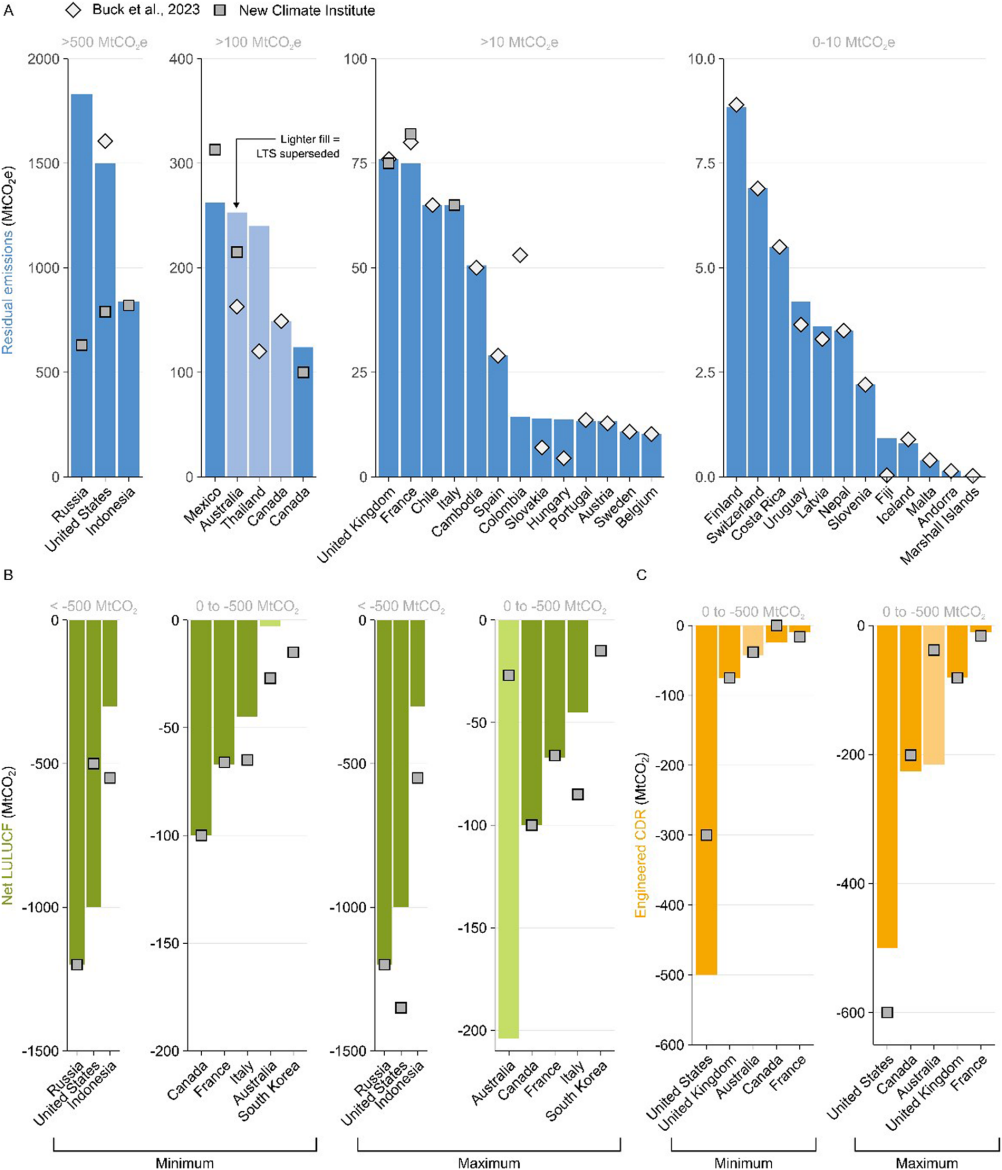
Comparisons between the LTS-SP dataset and Buck *et al*., 2023 and New Climate Institute’s assessment of G20 strategies. (**A**) details residual emissions. Figure split into four size classes depending on the magnitude of minimum residual emissions. Buck *et al*., 2023 depicted with a diamond, New Climate Institute’s G20 data with a square. Bars in lighter shades are superseded LTS, that is, they are no longer the most recent LTS for the country detailed. (**B**) details net LULUCF, split into minimum and maximum within New Climate Institute’s G20 assessment and by size class. C) depicts removals from engineered CDR, split into minimum and maximum within New Climate Institute’s G20 assessment and by size class.

Figure 7A shows a comparison between the minimum emissions within the LTS-SP dataset and minimum residual emissions detailed in Buck *et al*., 2023 and New Climate Institute’s assessment of G20 strategies. The LTS-SP dataset shows good agreement with Buck *et al*., 2023, with 17 of the 28 countries compared within ±5%. Several countries show wide disparities in estimates. We investigate all cases outside ±5%, correcting our dataset if necessary, or otherwise explaining the disparity in estimates in Table S2, found in Supplementary Information. The LTS-SP dataset shows less agreement with New Climate Institute’s assessment of G20 strategies, with only 3 of the 9 countries compared within a range of ±5%. As with Buck *et al*., 2023, we investigate all cases outside ±5%, explaining disparities in estimates in Table S2. We stress that disparities between estimates do not suggest errors, but rather different interpretations of the available data. Many disparities, for example, for Australia, Canada, Fiji, France, and Hungary, are explained by both Buck *et al*., 2023 and New Climate Institute’s assessment including removals, from engineered CDR or LULUCF, within larger sectoral totals. We use various methods and available data within the strategy to adjust for these cases.

Figure 7B shows a comparison between the minimum and maximum LULUCF for the LTS-SP dataset and the New Climate Institute’s assessment of G20 strategies. For minimum LULUCF estimates, 3 of the 8 countries compared are within ±5%. The same applies for maximum LULUCF estimates. We explain disparities in Table S3. Figure 7C details a comparison between the minimum and maximum engineered CDR removals for the same two datasets. For both minimum and maximum comparisons, only 1 country of the 5 compared is within ±5%, the UK. We explain all disparities in Table S3. Many disparities, for example, for Canada, France, Indonesia, and Italy are explained by New Climate Institute’s assessment including positive emissions within LULUCF or engineered CDR totals, or not differentiating between LULUCF and engineered CDR where this may be warranted.

## Usage Notes

On the practical use of the LTS-SP dataset, we end with a caveat on the current challenges of estimating LULUCF, before detailing four factors that may alter either the status of an LT-LEDS or the relevance of the scenarios and pathways they present, namely; a change in political mandate, the upgrading of a long-term target, changes in models or modelling practice, and recalculations within NGHGIs - commonly used as input into scenario modelling. We illustrate each with examples.

**Fig. 8:**
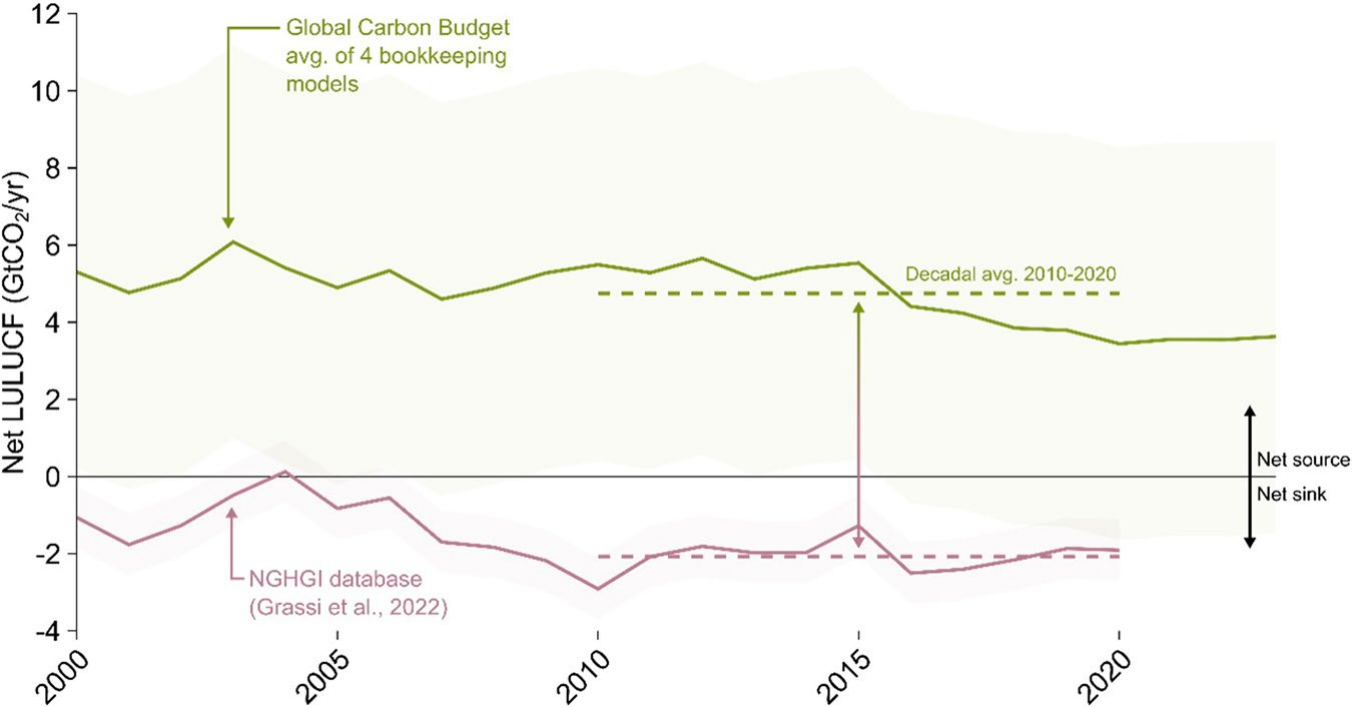
Comparison of net LULUCF emissions between the average of four bookkeeping models used by the Global Carbon Budget and the NGHGI database. Shading indicates 95% confidence interval. Data from refs. ^75,76^.

Firstly, LULUCF estimates within the LTS-SP dataset are not adjusted to align with estimates used in IPCC Assessment Reports. LULUCF emissions and removals should represent only those arising from anthropogenic activities, not natural disturbances or indirect effects (such as increased CO_2_ fertilisation or nitrogen deposition)^69^. Separating these in practice, however, remains a challenge^70–72^. Estimates within the LTS-SP dataset are likely based on the methodologies that support NGHGIs. In NGHGIs, the 2006 IPCC Guidelines estimate emissions and removals occurring on ‘*managed land*’, which is used ‘*as a proxy for anthropogenic effects*’^69^. This proxy means LULUCF estimates within NGHGIs capture, by definition, those emissions and removals arising from anthropogenic activities, but also natural and indirect effects^69^. Whilst countries are not required to report emissions and removals on ‘*unmanaged land*’, nearly all countries simply designate all land as managed, exacerbating these issues^73,74^. Within IPCC Assessment Reports, bookkeeping models and dynamic global vegetation models are used to isolate anthropogenic activities from these wider effects^72^. This leads to large disparities between global estimates. For the most recent decade for which data is available, 2010–2020, global LULUCF estimates as a mean of four bookkeeping models was a net source of 4.7 ± 5.1 (95% confidence interval) GtCO_2_/year^75^, whereas the combined global total from NGHGIs presents a net sink of −2.1 ± 0.8 GtCO_2_/year^76^ (see Fig. 8). Both estimates of LULUCF are highly uncertain when compared to other emission sources. The LTS-SP dataset, therefore should not be directly compared to benchmarks within the IPCC Assessment Reports, such as those derived from scenarios assessed by the IPCC, without first adjusting estimates to reconcile these disparities, ensuring like-for-like comparisons^72^.

LT-LEDS are large undertakings for national governments and can take several years to produce^77^. Though most long-term strategies within our sample are recent, 58 of the 80 strategies were published between 2021–2024, it is unlikely that the LTS-SP dataset will keep pace with political developments. Changes in government may alter a government’s mandate towards climate action, meaning scenarios presented in the government’s LT-LEDS may no longer be representative. For example, Australia’s first LT-LEDS was published in 2021 under the then centre-right Morrison government^78^. The Morrison government was a notable laggard in climate policy^79^, which manifested in Australia’s LT-LEDS through reducing the ambition of scenarios detailed within the strategy^78^. The main scenario presented, ‘*The Plan*’, was purposefully limited to an 85% emission reduction upon 2005 GHG emissions, despite Australia’s wider net zero GHG target and the modelling of four net zero scenarios excluded from the main text of the strategy^78,80^. The remaining 15% of the necessary mitigation was to be met by ‘*further technology breakthroughs*’, abdicating responsibility for these emissions^78,80^. The Morrison government was replaced by the centre-left Albanese government in 2022. The Albanese government has since submitted the ‘*2023 Annual Climate Change Statement*’ as a temporary LT-LEDS, with a fuller ‘*Net Zero Plan*’ to be submitted^81^. The Annual Climate Change Statement represents a domestic reporting obligation laid out in Australia’s 2022 Climate Change Act, to report on progress to the Australian Parliament, detailing near-term plans^82^. It therefore presents no scenarios to 2050, nor scenarios that model the needs of net zero. Nevertheless, the Albanese government has requested this document replace the 2021 LT-LEDS as the 2021 strategy ‘*reflected the approach of the previous government and is no longer aligned with the increased ambition of the Australian Government’s legislated commitments and our extensive emission-reduction action*s’^81^. A renewed political mandate can therefore alter the relevance of an LT-LEDS despite no material change to the country’s long-term target.

A renewed political mandate, however, can also be reflected by upgrading a long-term target. Changes to the long-term target can occur without requiring a country to revise its LT-LEDS, meaning, in the interim between strategies, scenarios may no longer be representative of long-term targets. For example, Thailand has submitted two LT-LEDS, the first in 2021, prior to COP26, and the second in 2022, during COP27^83,84^. The first, the ‘*Mid-century, Long-term Low Greenhouse Gas Emission Development Strategy*’ had a long-term target of net zero CO_2_ in 2065, with multiple scenarios^83^. The second LT-LEDS, ‘*Thailand’s Long-term Low Greenhouse Gas Emission Development Strategy*’ increased Thailand’s ambition by pledging to reach net zero GHGs in 2065, presenting revised scenario modelling^84^. In this case, upgrades to the target were communicated within LT-LEDS, leaving no gap between announcements and revised scenarios, however, parties to the Paris Agreement can communicate long-term targets by multiple means, including within NDCs. In these cases, the long-term target may be updated but the LT-LEDS not revised, reducing the relevance of any scenarios detailed therein.

Changes in models and modelling practice may also impact upon the relevance of scenarios. Canada’s latest LT-LEDS, for example, includes four scenarios that all attain Canada’s long-term target, net zero GHGs by 2050^85^. Using three models, two integrated assessment models and one computable general equilibrium (CGE) model, the LT-LEDS provides ranges for sectoral emissions in 2050^85^. Despite setting out a series of detailed technology assumptions, across all scenarios ‘*LULUCF was standardized to remove 100 MtCO*_*2*_*e by 2050*’, allowing ‘*more residual emissions to appear across the economy in the modelling*’^85^. This is a contested assumption considering that bioenergy is also modelled, impacting upon emissions and removals in LULUCF if the necessary biomass is domestically sourced^85^. This assumption means that the modelling ‘*does not take into account changing pressures on the land base from other sectors and does not necessarily account for activities on the land base that will be necessary to support a pathway to net-zero emissions*’, as acknowledged in the strategy^85^. Canada’s LT-LEDS similarly acknowledges the need to develop new methodologies and modelling to account for this limitation^85^.

Many LT-LEDS use NGHGIs as base years for long-term targets or scenarios and pathways. NGHGIs, however, are recalculated across all historic years in each submission to the UNFCCC^45^. Revisions may be necessary to incorporate methodological improvements or revisions to the activity data upon which emissions and removals are based^86^. Recalculations pose the greatest challenge to non-Annex I countries, which typically have more limited institutional capacity and therefore lean more heavily on international support^46^. NGHGIs for non-Annex I countries, therefore, frequently change with more limited documentation detailing recalculations. Whilst multiple historical emissions datasets exist to provide estimates for non-Annex I countries using consistent methods^87^, these estimates may differ to those used by the country within their LT-LEDS. Users of the dataset should therefore be cautious when comparing scenario and pathway estimates to historical emissions.

These four factors are not isolated to LTS, changes in political mandate and historical calculations both impact upon the relevance of reporting obligations such as NDCs. The challenges within LULUCF similarly impact upon the assessment of NDCs and their comparison to benchmarks in scenarios assessed by the IPCC. Nevertheless, these factors warrant attention when using the LTS-SP dataset. For example, the relevance of LT-LEDS can be determined by comparing the long-term targets to external sources, as demonstrated in the technical validation of this dataset. If using data for a single country or a small sub-sample, it should be possible to attain whether any changes in political mandate have occurred, though this can require detailed knowledge of domestic climate policy arrangements. Comparison to scenario benchmarks found in IPCC Assessment Reports warrant a need to adjust LULUCF. Despite these issues, LT-LEDS and EU LTS form the most readily available and comparable reporting provisions to understand how countries plan to attain their long-term targets^88^.

## Supplementary information

Supplementary Information

## Data Availability

No custom code was used in the creation of the dataset.
