# Supplementary material for: A dataset of emissions and removals from scenarios and pathways within long-term national climate strategies – the LTS-SP dataset
Source: Sci Data. 2025 Mar 23;12:485. doi: 10.1038/s41597-025-04804-4 (PMC11931002; doi:10.1038/s41597-025-04804-4)
Supplement: Supplementary file 1 — Supplementary Information [file 41597_2025_4804_MOESM1_ESM.docx]

A dataset of emission scenarios and pathways within long-term low emission development strategies – the LTS-SP dataset

Supplementary Information

Harry B. Smith^1,2^, Naomi E. Vaughan^1,2^ and Johanna Forster^2,3^

^1^School of Environmental Sciences, University of East Anglia, Norwich, NR4 7TJ, United Kingdom.
^2^Tyndall Centre for Climate Change Research, University of East Anglia, Norwich, NR4 7TJ, United Kingdom.
^3^School of Global Development, University of East Anglia, Norwich, NR4 7TJ, United Kingdom.

Detailed comparison of emissions & removals for validation datasets

Table S1. Ticks (✓) refer to the inclusion of the element for at least one scenario. Crosses (×) refer to the element not being available within the strategy analysed. Dashes (-) are used for engineered CDR, owing to their recent introduction into national climate policy, meaning the inclusion of engineered CDR can not necessarily be anticipated for every country, unlike LULUCF.

| Country | Total GHG emissions (excl. LULUCF) | Sectoral GHG emissions | LULUCF | Engineered CDR |
| --- | --- | --- | --- | --- |
| Andorra | ü | × | ü | - |
| Argentina | × | × | × | - |
| Armenia | ü | ü | ü | - |
| Australia | × | × | × | × |
| Austria | ü | ü | ü | ü |
| Belgium | ü | ü | × | - |
| Belize | ü | ü | ü | - |
| Benin | × | × | × | - |
| Bhutan | ü | × | ü | - |
| Bosnia and Herzegovina | ü | ü | ü | - |
| Bulgaria | ü | ü | ü | ü |
| Cambodia | ü | ü | ü | - |
| Canada | ü | × | ü | ü |
| Chile | ü | × | ü | - |
| China | × | × | × | - |
| Colombia | ü | × | ü | - |
| Costa Rica | ü | ü | ü | - |
| Croatia | ü | × | ü | - |
| Cyprus | ü | ü | ü | - |
| Czechia | × | × | × | - |
| Denmark | × | × | × | - |
| Equatorial Guinea | × | × | × | - |
| Ethiopia | ü | ü | ü | - |
| Estonia | ü | ü | ü | - |
| Fiji | ü | ü | ü | - |
| Finland | ü | ü | ü | ü |
| France | ü | ü | ü | ü |
| Gambia | ü | ü | ü | - |
| Georgia | ü | ü | ü | - |
| Germany | × | × | × | - |
| Greece | ü | ü | × | ü |
| Guatemala | ü | ü | ü | - |
| Hungary | ü | ü | ü | ü |
| Iceland | ü | × | ü | - |
| India | × | × | × | - |
| Indonesia | ü | ü | ü | - |
| Ireland | ü | ü | × | ü |
| Italy | ü | ü | ü | ü |
| Japan | × | × | × | - |
| Kazakhstan | ü | × | ü | - |
| Latvia | ü | × | ü | - |
| Lithuania | × | × | × | - |
| Luxembourg | × | × | × | - |
| Malta | ü | × | × | - |
| Marshall Islands | ü | ü | × | - |
| Mexico | ü | × | ü | - |
| Morocco | × | × | × | - |
| Nepal | ü | ü | ü | - |
| Netherlands | × | × | × | - |
| New Zealand | × | × | × | - |
| Nigeria | ü | ü | ü | - |
| North Macedonia | ü | ü | ü | - |
| Norway | × | × | × | - |
| Oman | ü | ü | × | ü |
| Panama | ü | ü | ü | - |
| Portugal | ü | ü | ü | - |
| Romania | ü | ü | ü | - |
| Russia | ü | × | ü | - |
| Serbia | ü | ü | ü | - |
| Singapore | × | × | × | - |
| Slovakia | ü | ü | ü | - |
| Slovenia | ü | ü | ü | - |
| Solomon Islands | ü | ü | ü | - |
| South Africa | × | × | × | - |
| South Korea | × | × | × | - |
| Spain | ü | ü | ü | - |
| Sri Lanka | × | × | × | - |
| Sweden | ü | ü | ü | - |
| Switzerland | ü | ü | ü | ü |
| Thailand | ü | ü | ü | - |
| Tonga | × | × | × | - |
| Tunisia | ü | ü | ü | - |
| Turkey | × | × | × | - |
| Ukraine | ü | × | ü | - |
| United Arab Emirates | ü | ü | × | ü |
| United Kingdom | ü | ü | × | ü |
| United States | ü | × | ü | ü |
| Uruguay | ü | ü | ü | - |
| Vanuatu | ü | ü | ü | - |
| Zimbabwe | ü | ü | ü | - |

Table S2. Assessment of the difference between minimum residual emission estimates between LTS-SP dataset and prior published data. Percentage difference from LTS-SP dataset shown.

| Country | Difference | Explanation |
| --- | --- | --- |
| Australia | LTS-SP dataset:  253 MtCO_2_e  Buck et al., 2023:  163 MtCO_2_e  (36% lower)  New Climate Institute:  215 MtCO_2_e  (15% lower) | Buck et al., 2023 uses 2005 values for the historic GHG inventory, found in Figure 1.4, Page 30. This is combined with the maximum reduction potentials from 2005-2050 shown in Figure 1.7, Page 36, for all totals shown in Figure 1.4, excluding LULUCF.  Table 6, Page 59 of Australia’s LT-LEDS Modelling and Analysis annex, shows the emissions prior to BECCS, DACCS, land-use and the inclusion of any international offsets for all scenarios. The minimum residual emissions at the point of net zero GHGs, is 200 MtCO_2_e, shown for the ‘NZE 100%’ scenario. This is different to ‘The Plan’ scenario, which appears to be the basis of Figure ES.2, Page 15 (as it maintains a 15% gap prior to reaching net zero GHGs, as detailed in Table 5, Page 58 of Australia’s LT-LEDS Modelling and Analysis annex). This poses a problem as to whether the main scenario, as presented in the strategy, should be used, despite the scenario failing to meet Australia’s long-term target, or whether scenarios that do reach this target but are deprioritised and shown only in additional documents, should be used instead. Given Buck et al., 2023 intends to examine ‘residual emissions at net zero’, ‘The Plan’ scenario falls short of this definition. Nevertheless, Table 6 of the Annex suggests the residual emissions in 2050 excluding sources of removals or international offsets, for ‘The Plan’ scenario totals 253 MtCO_2_e, substantially higher than the estimate derived from Figure 1.4 and Figure 1.7.  New Climate Institute’s assessment of G20 strategies appears to use numbers for ‘The Plan’ scenario, detailed in Table 6, Page 59, but use the row ‘Emissions before offsets’ as opposed to ‘gross emissions’ within the LTS-SP dataset. ‘Emissions before offsets’ already incorporates 38 MtCO_2_ of removals from BECCS, as noted in the ‘Negative emissions (BECCS)‘ row of Table 6. We exclude removals from BECCS from our calculation of residual emissions.  Note that we compare the 2021 LT-LEDS, now superseded by the more recent LT-LEDS in January 2024. |
| Canada | LTS-SP dataset:  124 MtCO_2_e  New Climate Institute:  100 MtCO_2_e  (19% lower) | The LTS-SP dataset uses Canada’s ‘Exploring Approaches for Canada’s Transition to Net-Zero Emissions’, published as an LT-LEDS in October 2022. We use values for all scenarios presented in Figure 1, Figure 4, Figure 7 and Figure 10, corresponding to the scenarios detailed in Table 4, Page 34. As Table 4 combines removals from BECCS in within Electricity and Industry sector totals, we use the ranges stated on Pages 22, 25, 28 and 31 to determine residual emissions, excluding BECCS, DACCS and LULUCF, leading to a minimum of 124 MtCO_2_e (Page 25 and Page 28, for the High Electrification Scenario and the High Use of Renewable and Alternative Fuels Scenario respectively).  New Climate Institute’s assessment of G20 strategies appears to use the totals found in Table 4, Page 34 of the 2022 strategy. Here the row ‘Total (excluding DAC and LULUCF)’ includes, as noted above, removals from BECCS. We exclude removals from BECCS from our calculation of residual emissions by using the ranges stated on Pages 22, 25, 28 and 31. |
| Colombia | LTS-SP dataset:  14 MtCO_2_e  Buck et al., 2023:  53 MtCO_2_e (268% higher) | Colombia has a goal of becoming ‘a carbon neutral country by 2050’, as stated on Page 19. Page 30 states that ‘To comply with what is required by science (1.5 to 2 °C), by 2050 Colombia needs to reduce GHG emissions by around 90% compared to 2015 emissions and balance the remaining 10% with proportional national absorptions (10%), to achieve a net zero balance between emissions and absorptions of greenhouse gases (carbon equivalents) from the year 2050’ [translation]. This implies a 90% trajectory for emissions reductions. This is similarly supported by Figure 20, Page 121, and the text shown on Page 120, which details that ‘Regarding the emission trajectories of the modelled scenarios, the grey area corresponds to a space of emissions that would allow Colombia to become carbon neutral, and for multiple scenarios it is necessary to achieve additional compensation to those already considered in 2050 (figure twenty). In particular, the scenarios lead to 90% decarbonization relative to 2015 levels and a substantial reduction in non-CO_2_ GHGs.’ [translation].  Figure 20, Page 121, indicates a residual emission in 2050 of around 20 MtCO_2_eq. Table 4, Page 128, alongside Page 129, Figure 23, indicates that for the ‘carbon neutrality scenario’ 12 MtCO_2_ is anticipated from the energy sector. Other scenarios presented in Table 4 and Figure 23 pertain to the ‘National Energy Plan’ or ‘PEN’, representing different energy technologies seen in Table 3, Page 114. Figure 24, Page 130, indicates that emissions from electricity generation could be as low as approximately 0.5 MtCO_2_. Section 4.3.4, Page 131, describes ‘Options for increasing greenhouse gas absorptions by 2050’, including ‘non-AFOLU’ options. These, however, pertain to enhanced oil recovery or post combustion CCS, options that typically reduce emissions, not provide negative emissions. Table 5, Page 131, appears to analyse the potential of these options as opposed to their inclusion within a modelled scenario. Table 7, Page 136, details a series of three scenarios to increase absorptions or avoid emissions in the AFOLU sector. For rows labelled absorptions, these total -54.79 MtCO_2_/yr in 2050 for Scenario 1, -164.8 MtCO_2_/yr in 2050 for Scenario 2, and -507.3 MtCO_2_/yr in 2050 for Scenario 3. Considering that Page 68 details that ‘Total absorptions’ in 2014 total -22.66 MtCO_2_, these scenarios represent at least double current absorptions, to upwards of 20 times. It is unclear whether these are part of modelled scenarios. Page 135 notes that ‘According to the results of the decarbonization scenarios for 2050, it would be necessary to capture from 14.4 to 31.5 MtCO_2_eq, depending on the levels of GHG emissions reduction achieved at the national level’.  Overall, our assessment of available data within Colombia’s LT-LEDS adds considerable ambiguity to our attempt to quantify residual emissions. Using values for 2014, found on Page 68, net emissions total 236.97 MtCO_2_e. a 90% reduction of this total would lead to a level of 23.7 MtCO_2_e, roughly in line with Figure 20. This value, however, includes emissions from land-use and excludes absorptions. This would, however, align with the current extent of absorptions from land-use, of around -22.66 MtCO_2_, reaching near net zero GHGs if this level is maintained. Excluding land-use emissions and applying the same 90% reduction would lead to a value of 15.8 MtCO_2_e remaining in 2050. Both values are within the range detailed on Page 135. Crucially, both values, alongside the range presented on Page 135 are greatly exceeded by the values for AFOLU absorption measures found in Table 7, Page 136. Given the ambiguity presented in the LT-LEDS, we use the range noted in Page 135 as indicative of residual emissions, leading to a minimum of 14.4 MtCO_2_e.  Buck et al., 2023 appears to use the ‘PEN disruption’ scenario from Table 4, Page 128 and shown in Figure 23, Page 129 (and first shown on Page 115, as detailed in Buck et al., 2023 Supplementary Data). This would appear to be a scenario in which the long-term target is not reached, as noted in Figure 20, Page 121, and Figure 23, Page 129. |
| Fiji | LTS-SP dataset:  0.93 MtCO_2_e  Buck et al., 2023:  0.04 MtCO_2_e (95% lower) | Buck et al., 2023 uses 2050 values found in Table 3, Pages 36-37, for the ‘Very High Ambition’ Scenario. As seen In Figure 7, Page 39 of Fiji’s LT-LEDS, this is the only scenario that reaches net zero GHGs prior to 2050, as per Fiji’s long-term target. Buck et al., 2023 sums the total for ‘Electricity and Other Energy Use’, ‘Domestic Air Transport’ and ‘Waste’, reaching 43,619 tCO_2_e in 2050, and excluding AFOLU.  The categorisation of AFOLU, however, combines agricultural emissions with land-use emissions and removals. AFOLU can be separated into agriculture and land-use via Table 35, Page 136. The LTS-SP dataset therefore separates and combines agricultural emissions for the scenario with the remaining totals in Table 3, in order to be consistent with other values. |
| France | LTS-SP dataset:  75 MtCO_2_e  Buck et al., 2023:  80 MtCO_2_e  (7% higher)  New Climate Institute:  82 MtCO_2_e  (9% higher) | Both studies appear to use the figure shown on Page 18 and replicated on Page 168, showing ‘Sinks and greenhouse gas emissions in France in 2050 according to the baseline scenario’ (Page 168).  We adjust for the avoided emissions from CCS, using the 6 MtCO_2_/year stated on Page 170, that appears to be represented as part of the ‘GHG sinks’ of the Figure on Page 168. This appears not to represent BECCS, unlike the remaining 10 MtCO_2_/year which is also detailed on Page 170 as BECCS and therefore thought to represent removals.  New Climate Institute’s assessment of G20 strategies appears to also use the figure first shown on Page 18, estimating the GHG sinks column as 82 MtCO_2_e. We make the same estimation but adjust for the avoidance of emissions from CCS on Page 170, as above. |
| Hungary | LTS-SP dataset:  13.8 MtCO_2_e  Buck et al., 2023:  4.5 MtCO_2_e (67% lower) | Buck et al., 2023 uses 2050 values found on Page 10, and graphically shown in Figure 2, Page 10. Here it is noted that ‘Natural sink capacities will be expanded to balance out the remaining emissions in 2050. It is forecasted in the EA and LA scenarios that 4.5 million tons of CO_2_eq/year will be naturally absorbed, mainly due to the increasing forest coverage’. Given Figure 2 shows only LULUCF as a net sink, it is reasonable to assume that the residual emissions shown for Energy, Agriculture and Waste management, shown on the positive y-axis, as an equivalent emission.  Figure 19, Page 41, however, details the balance of emissions within Energy, indicating around 10 MtCO_2_ is removed by ‘CCUS – Electricity Generation’ and ‘CCUS – Industry’ combined. Owing to their representation on the negative y-axis, and later reference to the use of biomass (for example, Page 43), we assume these categories represent removals from engineered CDR methods, such as BECCS. We therefore adjust the Energy sector total accordingly. |
| Iceland | LTS-SP dataset:  0.8 MtCO_2_e  Buck et al., 2023:  0.9 MtCO_2_e  (13% higher) | Both datasets use Figure 10, Page 47, to determine residual emissions. We note that Scenario E is slightly lower than Scenarios A and C. We therefore estimate this as a 100 ktCO_2_e lower than A and C. Given the legibility of Figure 10, it is reasonable to anticipate a difference of this magnitude. |
| Latvia | LTS-SP dataset:  3.6 MtCO_2_e  Buck et al., 2023:  3.3 MtCO_2_e (8% lower) | Latvia present a single 'With current measures' scenario, which considers only current policy and measures and the scenario only provides for the currently available commercial technologies (as shown in Figure 5, Page 26). It is not clear, however, if alternative trajectories have been produced, for example, on Page 26 it is noted that 'According to the initial indicative projections, the specified quantity of GHG emissions which will have to be compensated with removals in 2050 is approximately 3.6 million tCO_2_ eq'. This value would appear to be linked to modelling by an external and separate organisation. This differs from the level residual emissions shown in Figure 5 directly above this text, with a level well over 10 million t CO_2_ eq, including LULUCF as a net source. The trajectory depicted in red would appear to be a linear projection between targets for 2030 and 2040 and therefore is not a meaningful projection. In lieu of multiple scenarios, we use 3.6 MtCO_2_e as indicative of residual emissions.  Buck et al., 2023 uses the same reference seen on Page 26, but appears to note this as 3.3 MtCO_2_e as opposed to 3.6 MtCO_2_e as written. |
| Mexico | LTS-SP dataset:  262 MtCO_2_e  New Climate Institute:  313 MtCO_2_e  (19% higher). | Mexico presents a series of scenarios in relation to GHG emissions in Figure 21, Page 73. New Climate Institute’s assessment of G20 strategies uses the value shown for the ‘NDC more ambitious’ and ‘NDC policy’ scenario shown in the figure, for the year 2050. The LTS-SP dataset adjusts for the land-use CO_2_ emissions [labelled ‘CO2 Land Use Change’] included within this total, seen in Figure 22. |
| Russia | LTS-SP dataset:  1830 MtCO_2_e  New Climate Institute:  630 MtCO_2_e  (66% lower). | Russia's LT-LEDS presents two scenarios; the 'inertial scenario' and 'target (intensive) scenario'. The 'target (intensive) scenario' is the preferred scenario, as stated on Page 33 'As a basis for the implementation of the Strategy, it is proposed to use the target (intensive) scenario' and Page 15 'In view of the above, the inertial scenario is not considered as the main one. In order to fulfil the task set in the address of the President of the Russian Federation to the Federal Assembly of the Russian Federation dated April 21, 2021, and to minimize these risks, it is proposed to consider the target (intensive) scenario as the main one'.  The 'target (intensive) scenario' is thought to represent an 'additional policy' scenario, owing to the inclusion of 'additional emission reduction measures' (Page 15).  The results of each modelled scenario, in terms of the emissions for 2030 and 2050, can be found in the table on Page 34 (ANNEX). The results available, however, are not presented according to sectors, but only gross emissions, the net balance of 'adsorption', and the combined net emission balance. New Climate Institute’s assessment of G20 strategies appears to use the ‘net emissions’ row of the table shown on Page 34, which includes the ‘absorption’, thought to represent LULUCF. We use the gross emissions excluding LULUCF, labelled as ‘Greenhouse gas emissions’. |
| Slovakia | LTS-SP dataset:  14 MtCO_2_e  Buck et al., 2023:  7 MtCO_2_e (50% lower) | Buck et al., 2023 uses the ‘target emission gap’ referred to on Page 18 of Slovakia’s LT-LEDS. Modelled scenarios, however, detail that this gap is what remains after LULUCF removals are taken into account, leading to a 14 MtCO_2_e gap prior to accounting for removals (Page 15 & Figure 2, Page 15). Slovakia, however, notes that 7 MtCO_2_e represents a target to be reached, as this is the ‘amount that can potentially be eliminated through removals (the LULUCF sector)’ (Page 18), specifically via further policy measures (labelled as ‘NEUTRAL’ throughout sectoral chapters), that go beyond the modelled measures included in the WEM (with existing measures) and WAM (with additional measures) scenarios.  We prioritise the use of modelled scenarios to determine residual emissions, however it is also reasonable to use the target emission gap given this is stated as an objective to be reached. |
| Thailand | LTS-SP dataset:  240 MtCO_2_e (for the purpose of comparison)  Buck et al., 2023:  120 MtCO_2_e (50% lower) | Buck et al., 2023 appears to use the figures found on Page 44 of Thailand’s 2021 LT-LEDS, detailing trajectories for ‘carbon neutrality’ in 2065 or 2070. These trajectories, however, are for CO_2_ only.  The LTS-SP dataset uses ‘Thailand’s long-term low greenhouse gas emission scenario’ presented in ‘Figure 3-6’ on Page 38. This reaches net zero GHGs in 2090, but for comparison, we compare the emissions in 2065 to align with the timing of carbon neutrality seen on Page 44, and therefore the relevant reference for Buck et al., 2023.  Note that we compare the 2021 LT-LEDS, now superseded by the more recent LT-LEDS in November 2022. |
| United States | LTS-SP dataset: 1500 MtCO_2_e,  Buck et al., 2023: 1605 MtCO_2_e (7% higher)  New Climate Institute: 790 MtCO_2_e (47% lower). | The USA's LT-LEDS presents a total of 12 modelled scenarios across a range of technological assumptions. These are detailed in Table 1 on Page 22. Figure 3 presents a stepwise 'representative' emission reduction pathway with 7 alternative pathways explored. The representative pathways 'provides a rough approximation for reaching net-zero emissions using contributions from all sectors', as detailed on Page 22. The results of modelled pathways serve as modelled ranges across a series of figure supporting sector level analysis in Chapters 4-6. Figure 3's presentation, however, means residual emissions at the time of the net-zero target cannot be determined. Figure 17, on Page 45, provides a means of estimating residual emissions, however, only by approximation from the figure itself. Nor are specific values cited elsewhere within the text. Some limited detail of residual emissions in 2050 by gas, including fluorinated gases, can be found in Figure 12 and 13, Pages 36 and 37 respectively. These, however, do not appear to show differing levels, with Figure 13 implying residuals for nitrous oxide comparative to methane, however nitrous oxide is visibly lower in Figure 12. These estimates, therefore, cannot be used with accuracy to determine residual emissions. We therefore approximate residual emissions from the Figure 17 itself, using the central line within the ranges presented.  Buck et al., 2023 appears to also use Figure 17, Page 45, to estimate residual emissions, but also notes the ‘energy sector and non-CO2 GHG figure captions’. We take this to refer to Figure 13 and Figure 12, which are subject to the issues we outline above. We are unclear as to how the specific value has been reached, but note that a difference of a 100 MtCO_2_e is reasonable considering the legibility of Figure 17.  New Climate Institute’s assessment of G20 strategies appears to use the range presented in Figure 18, Page 48 for LULUCF, which is estimated to range between -500 to -1350 MtCO_2_e in 2050, where -500 MtCO_2_e is the upper range of the BAU scenario and -1350 MtCO_2_e the lower bound of the ‘NCS Action Range’. CDR (here referring to engineered CDR) is estimated to be -300 to -600 MtCO_2_e in 2050, leading to a total range of 800 MtCO_2_e. We believe this to be too low judging from Figure 17, perhaps owing to the inclusion of the BAU range in Figure 18, whereas LULUCF in Figure 17 may refer to the ‘NCS Action Range’ shown in Figure 18. Given the legibility of Figure 17, and the incorporation of ranges, such a difference in estimates should be anticipated. |
| Uruguay | LTS-SP dataset:  4.19 MtCO_2_e  Buck et al., 2023: 3.64 MtCO_2_e (13% lower) | Buck et al., 2023 uses Table 04, Page 55. This details only CO_2_ emissions from energy and industrial processes. For methane and nitrous oxide, Uruguay's LT-LEDS explores scenarios which stabilise emissions prior to 2050, detailed on Pages 62-70. We combine these same estimates with Table 04 to determine total GHG emissions in 2050. |

Table S3. Assessment of the difference between LULUCF and engineered CDR between the LTS-SP dataset and New Climate Institute’s assessment of G20 strategies. Percentage difference from LTS-SP dataset shown.

| Country | Element | Difference | Explanation |
| --- | --- | --- | --- |
| Australia | LULUCF | LTS-SP dataset: Max -204 MtCO_2_,  Min -3 MtCO_2_,  New Climate Institute:  -27 MtCO_2_ | Table 6, Page 59 of Australia’s LT-LEDS Modelling and Analysis annex, shows the emissions prior to BECCS, DACCS, land-use and the inclusion of any international offsets. The minimum LULUCF at the point of net zero GHGs, is -3 MtCO_2_e, shown for the ‘NZE no offsets’ scenario. The maximum engineered CDR at the point of net zero GHGs, is -204 MtCO_2_e, shown for the ‘NZE no trade’ scenario, referring to the row ‘Domestic offsets: Land sector’.  New Climate Institute’s assessment of G20 strategies appears also to use Table 6, Page 59, but for ‘The Plan’ scenario, which incorporates 27 MtCO_2_ of removals from the ‘Land sector’, as noted in Table 6. We note that ‘The Plan’ scenario does not reach net zero GHGs, as per the ‘NZE’ scenarios in Table 6. We therefore use the NZE scenarios as representative of Australia’s LULUCF.  Note that we compare the 2021 LT-LEDS, now superseded by the more recent LT-LEDS in January 2024. |
| Indonesia | LULUCF | LTS-SP dataset:  -300 MtCO_2_,  New Climate Institute:  -550 MtCO_2_ | New Climate Institute’s assessment appears to use Figure 6, Page 37, to determine LULUCF, estimating the amount of removals by the extent of the negative y-axis for LCCP scenario in 2050. Figure 6 details emissions for AFOLU under both ‘CPOS and ‘LCCP’ scenarios and includes not only positive emissions from agriculture on the positive y-axis, but similarly emissions from land-use.  When combined to reach a net total, the net FOLU total in 2050 is near -300 MtCO_2_, as seen in Figure 5, Page 34. We therefore use Figure 5 to determine the contribution from FOLU. |
| Italy | LULUCF | LTS-SP dataset:  -45 MtCO_2_,  New Climate Institute:  Min -65 MtCO_2_  Max -85 MtCO_2_ | New Climate Institute’s assessment appears to use the range stated on Page 19 ‘These reduction dynamics, however intense, leave a quantity of emissions to be compensated of approximately 65-85 Mton CO2 eq.’ [translated]. It is then thought to be assumed that this amount is compensated by LULUCF, however, Page 19 also notes that ‘the absorption capacity of the LULUCF sector must be considered which, with adequate policies to fight fires and sustainable soil management, can be brought back to an all-time high (equal to 45 Mton CO2 eq)’ [translation], and therefore doesn’t appear to compensate for the total extent of residual emissions. This is confirmed by Figure 3.5.4, Page 83. We note that Page 19 also details the potential use of BECCS, similarly included in Figure 2.2.4, Page 35.  If residual emissions equate to at least 65 MtCO_2_eq, as stated on Page 19, then emissions from known sectors stand at 50 MtCO_2_eq (22 MtCO_2_e [Industry, including combustion, text on Page 49 and Figure 3.1.6] + 23 MtCO_2_e [Agriculture, Figure 3.4.3, Page 76] + approximately 5 MtCO_2_eq [Waste, Figure 1.3.2, Page 19]). Subtracting the maximum extent of LULUCF, stated on Page 19 and displayed Figure 3.5.4, Page 83, of 45 MtCO_2_eq, leaves 5 MtCO_2_eq remaining. Given that Figure 1.3.2, Page 19, shows that ‘energy industries [Industrie energetiche]’ to be net-negative at approximately -5 MtCO_2_e [a similar extent to Waste judging by the Figure 1.3.2], it serves that, if BECCS was deployed at a scale of -20 MtCO_2_eq, as implied by Page 19, positive emissions for the ‘energy industries’ would equate to 15 MtCO_2_eq, approximately the extent seen in Figure 2.2.4, Page 35. We therefore use 15 MtCO_2_eq as our estimate for ‘energy industries’. This would then total 65 MtCO_2_e. It is unclear how the 85 MtCO_2_e, detailed on Page 19, can be reached using data presented within the strategy. We therefore discount a scenario with a residual of this amount. We assume this amount is exclusive of avoided emissions from carbon capture and storage, as implied on Page 19. |
| South Korea | LULUCF | LTS-SP dataset: Not provided,  New Climate Institute: -15MtCO_2_. | New Climate Institute’s assessment appears to use Figure 4-30, Page 99, to determine LULUCF. This shows a decline in removals from forests through to 2050. This appears to reflect only removals not emissions and removals combined, as Figure 4-31, Page 98, details removals at ‘45.7 million ton CO_2_eq’ in 2017, whereas Table 2-1, Page 30 and 31, details total LULUCF as -41.6 MtCO_2_. Figure 4-30 appears to show a forward-looking projection of the same variable as Figure 4-31. This projection represents existing policy, a trend which South Korea seeks to reverse via the policies outline in Section 7.2, beginning on Page 99. We therefore view this projection as partial, excluding emissions, and representative of business as usual, as opposed to the policies proposed. As a result, we do not recommending using this figure to determine LULUCF. |
| Australia | Engineered CDR | LTS-SP dataset: Max -216 MtCO_2_, Min -43 MtCO_2_,  New Climate Institute: -38 MtCO_2_ (12-82% higher). | Table 6, Page 59 of Australia’s LT-LEDS Modelling and Analysis annex, shows the emissions prior to BECCS, DACCS, land-use and the inclusion of any international offsets. The minimum engineered CDR at the point of net zero GHGs, is -43 MtCO_2_e, shown for the ‘NZE 100%’ scenario. The maximum engineered CDR at the point of net zero GHGs, is -216 MtCO_2_e, shown for the ‘NZE no offsets’ scenario, combining rows for ‘Negative emissions (BECCS)’ and ‘Negative emissions (DAC)’. We assume DAC represents permanent removals and not the use of direct air capture for CO_2_ utilisation that results in short-term storage.  New Climate Institute’s assessment of G20 strategies appears also to use Table 6, Page 59, but for ‘The Plan’ scenario, which incorporates 38 MtCO_2_ of removals from BECCS, as noted in the ‘Negative emissions (BECCS)‘ row of Table 6. We note that ‘The Plan’ scenario does not reach net zero GHGs, as per the ‘NZE’ scenarios in Table 6. We therefore use the NZE scenarios as representative of Australia’s engineered CDR.  ‘The Plan’ scenario, appears to be the basis of the figure depicting the main scenario, Figure ES.2, Page 15 of Australia’s LT-LEDS (as it maintains a 15% gap prior to reaching net zero GHGs, as detailed in Table 5, Page 58 of Australia’s LT-LEDS Modelling and Analysis annex). This poses a problem as to whether the main scenario, as presented in the strategy, should be used, despite the scenario failing to meet Australia’s long-term target, or whether scenarios that do reach this target but are deprioritised and shown only in additional documents. |
| Canada | Engineered CDR | LTS-SP dataset: Max -226 MtCO_2_, Min -24 MtCO_2_,  New Climate Institute: Max -201 MtCO_2_, Min 0 Mt CO_2_ (11-100% higher). | New Climate Institute’s assessment of G20 strategies appears to use the totals found in Table 4, Page 34 for ‘DAC’, ranging from 0 to -201 MtCO_2_. This excludes removals from BECCS which are combined within Electricity and Industry sector totals within Table 4. We calculate a engineered CDR total for the scenarios in Table 4 by using the ranges stated on Pages 22, 25, 28 and 31, which are inclusive of ‘LULUCF, BECCS, and DAC’. As the contribution from LULUCF remains constant across the scenarios, as noted on Page 19 (‘Across all scenarios, the modelling approach for Canada’s LTS assumes a contribution of -100 Mt from LULUCF’), the contribution from engineered CDR can be determined by subtracting this contribution from the ranges detailed. |
| France | Engineered CDR | LTS-SP dataset: -10 MtCO_2_,  New Climate Institute: -16 MtCO_2_ (60% lower). | New Climate Institute’s assessment of G20 strategies appears to use the figure shown on Page 18 and Page 168, estimating the GHG sinks column as 82 MtCO_2_, with a contribution of 16 MtCO_2_ from ‘Carbon Capture and Storage’, labelled by New Climate Institute as ‘tech CDR’. We make the same estimation, but adjust for the avoided emissions from CCS, using the 6MtCO_2_/year stated on Page 170, that appears to be represented as part of the ‘GHG sinks’ of the Figure on Page 168. This appears not to represent BECCS, unlike the remaining 10 MtCO_2_/year which is also detailed on Page 170 as BECCS and therefore thought to represent removals. |
| USA | Engineered CDR | LTS-SP dataset: Max -1500 MtCO_2_, Min -1500 MtCO_2_,  New Climate Institute: Max -300 MtCO_2_, Min -600 Mt CO_2_ (40% lower – 20% higher). | New Climate Institute’s assessment appears to use the range presented in Figure 17, Page 45 estimating the range seen for ‘CDR’ to be -300 to -600 MtCO_2_ in 2050. We approximate the contribution from CDR similarly from Figure 17 but use the central line within the ranges presented. Given the legibility of Figure 17, and the incorporation of ranges, such a difference in estimates should be anticipated. |
